# Dissection of complicate genetic architecture and breeding perspective of cottonseed traits by genome-wide association study

**DOI:** 10.1186/s12864-018-4837-0

**Published:** 2018-06-13

**Authors:** Xiongming Du, Shouye Liu, Junling Sun, Gengyun Zhang, Yinhua Jia, Zhaoe Pan, Haitao Xiang, Shoupu He, Qiuju Xia, Songhua Xiao, Weijun Shi, Zhiwu Quan, Jianguang Liu, Jun Ma, Baoyin Pang, Liru Wang, Gaofei Sun, Wenfang Gong, Johnie N. Jenkins, Xiangyang Lou, Jun Zhu, Haiming Xu

**Affiliations:** 1 Institute of Cotton Research of Chinese Academy of Agricultural Sciences (ICR, CAAS), State Key Laboratory of Cotton Biology, Key Laboratory of Cotton Genetic Improvement, Ministry of Agriculture, Anyang, 455000 People’s Republic of China; 2 Institute of crop science and Institute of Bioinformatics, College of Agriculture and Biotechnology, Zhejiang University, Hangzhou, 310058 People’s Republic of China; 3 Shenzhen Huada Gene Research Institute, Shenzhen, 518031 People’s Republic of China; 4 Institute of industrial Crops, Jiangsu Academy of Agricultural Sciences, Nanjing, 210014 People’s Republic of China; 5 Economic Crop Research Institute, Xinjiang Academy of Agricultural Science, Urumqi, 830002 People’s Republic of China; 6 Department of Computer Science and Information Engineering, Anyang Institute of Technology, Anyang, 455000 People’s Republic of China; 7 USDA-ARS at Mississippi State, Jackson, MS 39762-5367 USA; 8 Department of Pediatrics, Biostatistics Division Arkansas Children‘s Hospital Research Institute School of Medicine, University of Arkansas for Medical Sciences, Little Rock, AR 72202 USA

**Keywords:** Complex traits, Cottonseed traits, Association mapping, GWAS, Gene by environment interaction, Epistasis, Mixed linear model, Molecular breeding by design

## Abstract

**Background:**

Cottonseed is one of the most important raw materials for plant protein, oil and alternative biofuel for diesel engines. Understanding the complex genetic basis of cottonseed traits is requisite for achieving efficient genetic improvement of the traits. However, it is not yet clear about their genetic architecture in genomic level. GWAS has been an effective way to explore genetic basis of quantitative traits in human and many crops. This study aims to dissect genetic mechanism seven cottonseed traits by a GWAS for genetic improvement.

**Results:**

A genome-wide association study (GWAS) based on a full gene model with gene effects as fixed and gene-environment interaction as random, was conducted for protein, oil and 5 fatty acids using 316 accessions and ~ 390 K SNPs. Totally, 124 significant quantitative trait SNPs (QTSs), consisting of 16, 21, 87 for protein, oil and fatty acids (palmitic, linoleic, oleic, myristic, stearic), respectively, were identified and the broad-sense heritability was estimated from 71.62 to 93.43%; no QTS-environment interaction was detected for the protein, the palmitic and the oleic contents; the protein content was predominantly controlled by epistatic effects accounting for 65.18% of the total variation, but the oil content and the fatty acids except the palmitic were mainly determined by gene main effects and no epistasis was detected for the myristic and the stearic. Prediction of superior pure line and hybrid revealed the potential of the QTSs in the improvement of cottonseed traits, and the hybrid could achieve higher or lower genetic values compared with pure lines.

**Conclusions:**

This study revealed complex genetic architecture of seven cottonseed traits at whole genome-wide by mixed linear model approach; the identified genetic variants and estimated genetic component effects of gene, gene-gene and gene-environment interaction provide cotton geneticist or breeders new knowledge on the genetic mechanism of the traits and the potential molecular breeding design strategy.

**Electronic supplementary material:**

The online version of this article (10.1186/s12864-018-4837-0) contains supplementary material, which is available to authorized users.

## Background

Cotton, one of the most important crops, has been used extensively in many fields, such as textiles, food consumption and medical use. Cottonseed accounts for approximately two-thirds of the total cotton harvested while the remaining one-third is explained by fiber [1]. Cottonseed meal is a very good source of protein, and generally less expensive per unit of protein than soybean meal [2]. Benefiting from effective decrease of free gossypol in cottonseed, cottonseed protein has been regarded as a good food source with well-balanced and high nutritional value [3]. Cottonseed oil is typically composed of saturated fatty acids (about 1% myristic acid (C14:0), 22% palmitic acid (C16:0), and 3% stearic acid (C18:0) which confer a relatively stable vegetable oil without partial hydrogenation, and enough unsaturated fatty acids (22% oleic acid (C18:1) and 52% linoleic acid (C18:2) which are requisite ingredients for a heart healthy oil [4]. In addition, cottonseed oil could also be purified to be a kind of alternative fuel for diesel engines [5].

Using linkage mapping, several studies have been carried out for detecting QTLs associated with cottonseed traits in specifically designed mapping populations [6–10]. However, limited recombination events and low genetic diversity in the designed population are major obstacles to distinguishing more QTLs at fine level by conventional linkage mapping [11]. The genome-wide association study (GWAS), as an alternative strategy, has been proved to be an effective way to identify genetic variants underlying traits at a relatively finer resolution in maize, rice, soybean, sesame, and other crops [11–13]. Badigannavar et al. used the GWAS successfully to detect genetic diversity, population structure and variants associated with cottonseed quality traits [14], but this study based on AFLP markers, and might lose precision and power due to relatively small number of markers. Currently, the availability of draft genome sequence of the G. *raimondii*, *G. arboreum* and the *G. hirsutum* provides a crucial groundwork for identification, isolation and manipulation of important cotton functional genes controlling agronomic, yield, and quality traits [15–20]. Due to the narrow genetic basis and the characteristic of allotetraploid genome, it is difficult to discover or design polymorphic molecular marker in cotton, however, as the development of high-throughput DNA sequencing technology, it has been possible to discover a large number of SNPs [21, 22] saturated in the entire cotton genome; as a result, GWAS could be conducted for exploring intricate genetic architecture of most cotton traits of interested, which is mostly referred to the underlying genetic basis and variation properties of a trait, and usually depicted by the associated QTLs and their genetic effects including additive, dominance, epistasis and their interactions with environments [23].

Generally, most GWAS approaches are based on simple additive or additive and dominance effects models [24, 25], ignoring the fact that both the polygenic interaction (epistasis) and the gene × environment interaction are involved in the genetic variation of a complex trait substantially; as a result, these methods are not effective for detecting many minor-effect loci and paired epistatic loci and will result in the problem of missing heritability [26–28]. Zeng et al. (2016) investigated the association between SNPs of *GhSus* family genes and fiber- and seed-related traits in 277 upland cotton accessions based on an epistatic association mapping (EAM) model, which included main-effects, epistatic effects and one-dimensional gene × environment interaction effect. However, their study only focused on some specific genes, it’s still very valuable to explore the whole genetic architecture of the cottonseed traits across entire AD genomes [29]. In this study, we employed a newly proposed method, which could analyze single-locus effects, epistatic effects, and their interaction effects with environments simultaneously, to conduct the GWAS for seven cottonseed traits (protein content, oil content, myristic acid, palmitic acid, stearic acid, oleic acid, and linoleic acid), based on the phenotype of 316 accessions in 3 different locations and the genotype of ~ 390 k SNPs. This study aims to uncover the complicate genetic architecture of cottonseed traits and predict the breeding potential of the detected genetic variants in genetic improvement of target traits.

## Results

### Population structure and LD analysis

According to the results of the population structure analysis, the total cotton accessions could be classified into two subgroups (Additional file 1: Figure S2). The half LD decay distance measured by the average correlation coefficient (*r*^2^) of pairwise SNPs decreases to the half of its maximum value (Additional file 2: Figure S3) was ~ 160 kb, in the range of maize (~ 500 kb) and rice (~ 123 kb in *Indica* rice landrace and ~ 167 kb in *Japonica* rice landrace) [30, 31], which is in concert with the result expected for cotton as a species of often cross-pollinated plant. Abdurakhmonov et al. reported that the genome-wide average of LD block size of cotton, based on SSR marker at *r*^2^ ≥ 0.1, is less than 4 Mb in the landrace germplasm and larger than 12 Mb in the improved variety germplasm [32]; whereas the corresponding value in our study was around 163 Kb, much higher resolution than those in the previous studies.

### Total heritability analysis for seven cottonseed traits

According to the result of SNP screening by GMDR method for each trait, total 4864 SNPs (996 SNPs for protein, 717 SNPs for oil, 784 SNPs for oleic acid, 889 SNPs for linoleic acid, 1026 SNPs for palmitic acid, 881 SNPs for myristic acid, and 1139 SNPs for stearic acid) were selected and used to perform the subsequent GWAS analysis. A total of 124 significant associated QTSs were identified for the protein content, the oil content, and the fatty acids. In general, all seven cottonseed traits exhibited significant additive and dominance effects; however, the epistatic effects and gene-environment effects, were largely diverse across traits (Table 1); the broad-sense heritabilities of these traits varied from 71.62 to 93.43%.

**Table 1:** Estimated heritability for seven cottonseed traits

Trait	${h}_A^2$	${h}_D^2$	${h}_{AA}^2$	${h}_{AD}^2$	${h}_{DA}^2$	${h}_{DD}^2$	${h}_{AE}^2$	${h}_{DE}^2$	${h}_{AAE}^2$	${h}_{ADE}^2$	${h}_T^2$
Protein	12.51	15.74	1.72	21.18	10.85	31.43	–	–	–	–	93.43
Oil	10.14	33.44	10.79	6.98	14.35	6.21	–	3.69	–	7.76	93.36
Palmitic	18.30	24.90	2.17	–	3.72	36.79	–	–	–	–	85.88
Linoleic	14.15	45.09	0.79	3.89	–	20.00	1.28	1.24	–	–	86.44
Oleic	23.13	54.99	2.69	–	–	–	0..92	–	–	–	81.73
Myristic	34.01	36.84	–	–	–	–	1.07	–	2.24	–	74.16
Stearic	19.79	23.37	–	–	–	–	25.05	3.41	–	–	71.62

${h}_A^2$, ${h}_D^2$, ${h}_{AA}^2$, ${h}_{AD}^2$, ${h}_{DA}^2$, and ${h}_{DD}^2$ are the heritabilities in percentage due to the additive effects, the dominant effects, the additive × additive epistasis effects, the additive × dominance epistasis effects, the dominance × additive epistasis effects and the dominance × dominance epistasis effects of all QTSs, respectively; ${h}_{AE}^2$, ${h}_{DE}^2$, ${h}_{AAE}^2$ and ${h}_{ADE}^2$ are the heritabilities in percentage due to the additive by environment interaction effects, the dominance by environment interaction effects, additive × additive epistasis by environment interaction effects, additive × dominance epistasis by environment interaction effects of all QTSs, respectively; ${h}_T^2$ is the total broad-sense heritability in percentage

The protein content was not only regulated by additive and dominance effects, but also by four kinds of epistatic effects (*aa, ad, da, dd*) with total epistatic heritability of 65.18%, in which the heritability of dominance × dominance epistatic effect (${h}_{DD}^2=31.43\%$) was larger than that of other epistatic effects. Similarly, the palmitic acid was affected by additive and dominance effects, as well as epistatic effects (*aa, da, dd*), and the dominance × dominance effect played a key role in genetic variation (${h}_{DD}^2=36.79\%$). Like the protein content, the oil content was also affected by all genetic main effects, but the dominance effect (${h}_D^2=33.44\%$) contributed the largest to the total heritability, and dominance × environment and additive-dominance epistasis × environment effects (*de* and *ade*) were also involved in the genetic architecture of the oil content. The oleic acid and the linoleic acid are two important and genetically related traits, and both were regulated mainly by dominance effect (${h}_D^2=54.99\%$ for the oleic acid, and ${h}_D^2=45.09\%$ for the linoleic acid); Furthermore, epistatic effects and gene × environment interaction effects were also involved in the genetic architecture of these two traits and account for more proportion of the genetic heritability for the linoleic acid than that for the oleic acid. The additive and dominance effects explained 70.85% of the total variation and 95.54% of total heritability for the myristic acid. On the stearic acid, the gene × environment interaction effects were very prominent, compared with the main effect (the additive effect); notably, the additive × environment interactions (${h}_{AE}^2=25.05\%$) exhibited remarkable effects on the stearic acid than the dominance × environment interactions (${h}_{DE}^2=3.41\%$).

### Genetic architecture of the protein content and the oil content

**Fig. 1:**
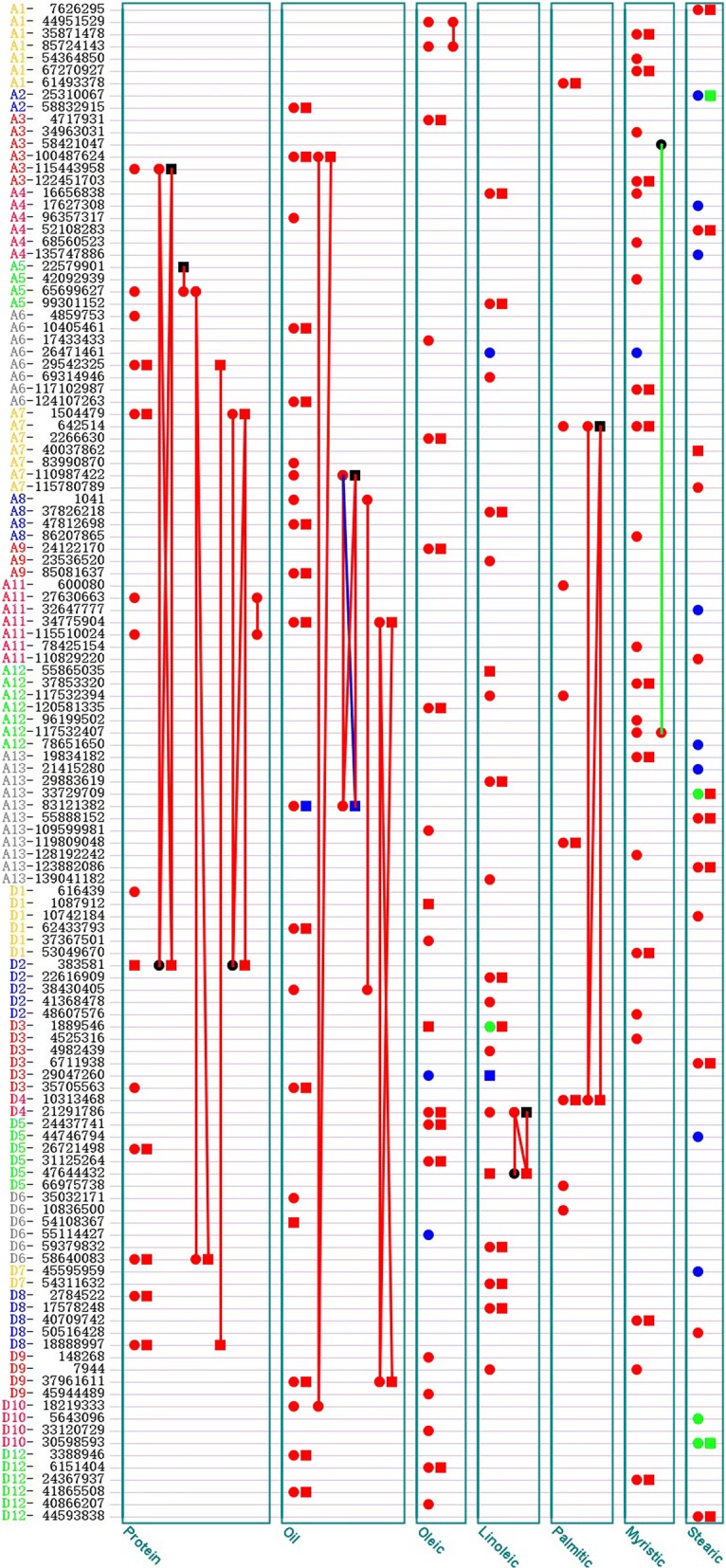
Network plot of highly significant QTSs associated with seven cottonseed traits. Red dot (square) indicates the QTS expresses additive (dominance) effects, green dot (square) indicates the QTS expresses additive (dominance) by environment interaction effect, blue dot (square) indicates the QTS expresses both additive (dominance) effect and additive (dominance) by environment effect, black dot (square) indicate the QTS doesn’t express additive (dominance) effect but interacts with other QTS, red line indicates there is only interaction (epistasis) between genetic components of two QTSs at the ends of the line, green line indicates there is only interaction between epistasis and environment for the ends of the line, blue line indicates there is both epistasis effect and interaction between epistasis and environment for the end of the line

15 QTSs, consisting of 5 QTSs with significant main-effects of additive or dominance, named individual QTS hereafter, and 6 pairs of QTSs involved in epistatic interaction, named epistatic QTSs hereafter, were detected to be significantly associated with the protein content (Table 2**,** Fig. 1 and Additional file 3: Table S3). QTS A3_115443958 was identified with the largest additive heritability (−log_10_*p* = 69.59, ${h}_a^2=5.14\%$). Of epistatic QTSs, three pairs of QTSs A3_115443958 & D2_383581, A6_29542325 & D8_18888997, and A7_1504479 & D2_383581, accounted for major epistatic heritability of 61.03%. Generally, QTSs involved in epistasis with heritability from 0.12 to 20.16%, also had both additive and dominance effects. In addition to the dominance effect, D2_383581 contributed four kinds of epistatic effects (*aa, ad, da, dd*) through interaction with A3_115443958, wherein the additive-dominance epistasis explained 20.16% heritability, and other 3 kinds of epistasis (*aa, da, dd*) taked effects on the protein together with A7_1504479.

**Table 2:** Genome-wide significant QTSs associated with the protein content and the oil content at -log_10_p > 7 (at least one kind of effect of QTS)

Trait	QTS	^a^Chr.	^b^Allele	^c^Effect type	Predicted effect	−log_10_*p*	*h*^*2*^(*%*)
Protein	A3_115443958	A3	T/A	*a*	1.302	69.59	5.14
	A6_29542325	A6	G/A	*a*	0.408	8.16	0.50
		A6	G/A	*d*	−0.477	1.33	0.34
	A7_1504479	A7	A/G	*a*	0.363	6.59	0.40
		A7	A/G	*d*	1.019	4.83	1.57
	A11_27630663	A11	A/G	*a*	0.535	14.64	0.87
	A11_115510024	A11	A/G	*a*	0.541	14.95	0.89
	D1_616439	D1	C/T	*a*	−0.909	32.58	2.50
	D2_383581	D2	A/C	*d*	−1.842	8.56	5.14
	D3_35705563	D3	T/C	*a*	−0.418	8.83	0.53
	D6_58640083	D6	G/A	*a*	0.323	5.36	0.32
		D6	G/A	*d*	−1.362	7.57	2.81
	D8_18888997	D8	G/A	*a*	0.38	7.23	0.44
		D8	G/A	*d*	−0.513	1.35	0.40
	A3_115443958 & D2_383581	A3 & D2	T/A & A/C	*aa*	−0.297	4.15	0.53
		A3 & D2	T/A & A/C	*ad*	−2.579	11.98	20.16
		A3 & D2	T/A & A/C	*da*	−0.784	4.74	1.86
		A3 & D2	T/A & A/C	*dd*	1.516	1.94	3.48
	A6_29542325 & D8_18888997	A6 & D8	G/A & G/A	*dd*	3.167	8.44	15.19
	A7_1504479 & D2_383581	A7 & D2	A/G & A/C	*aa*	−0.142	1.31	0.12
		A7 & D2	A/G & A/C	*da*	1.512	9.17	6.93
		A7 & D2	A/G & A/C	*dd*	−2.902	3.2	12.76
Oil	A2_58832915	A2	G/A	*a*	0.24	2.5	0.16
		A2	G/A	*d*	−0.854	7.79	0.99
	A6_124107263	A6	G/A	*a*	0.517	11.41	0.73
		A6	G/A	*d*	−1.277	5.81	2.22
	A7_110987422	A7	A/C	*a*	−0.256	3.25	0.18
	A9_85081637	A9	T/C	*a*	−0.555	13.04	0.84
		A9	T/C	*d*	0.563	1.47	0.43
	A13_83121382	A13	T/C	*a*	0.763	24.58	1.58
		A13	T/C	*d*	−3.057	18.52	12.71
		A13	T/C	*de2*	−1.956	3.25	5.20
		A13	T/C	*de3*	1.262	1.59	2.17
	D3_35705563	D3	T/C	*a*	0.163	1.57	0.07
		D3	T/C	*d*	−1.834	8.06	4.57
	D6_54108367	D6	G/A	*d*	1.639	8.42	3.65
	D10_18219333	D10	C/T	*a*	−0.98	40.02	2.61
	D12_3388946	D12	A/G	*a*	−0.405	7.39	0.45
		D12	A/G	*d*	−0.608	1.37	0.50
	D12_41865508	D12	G/A	*a*	0.526	12.01	0.75
		D12	G/A	*d*	0.911	2.49	1.13
	A3_100487624 & D10_18219333	A3 & D10	G/A & C/T	*aa*	−1.208	44.59	7.94
		A3 & D10	G/A & C/T	*da*	1.718	32.67	8.03
	A11_34775904 & D9_37961611	A11 & D9	G/A & C/T	*aa*	−0.565	12.61	1.74
		A11 & D9	G/A & C/T	*ad*	− 0.608	1.48	1.00
		A11 & D9	G/A & C/T	*da*	0.741	1.95	1.49
	A7_110987422 & A13_83121382	A7 & A13	A/C & T/C	*aa*	0.367	5.95	0.73
		A7 & A13	A/C & T/C	*ad*	−1.483	3.63	5.98
		A7 & A13	A/C & T/C	*da*	1.332	4.79	4.83
		A7 & A13	A/C & T/C	*dd*	2.138	3.1	6.21
		A7 & A13	A/C & T/C	*ade2*	−1.688	2.02	7.76

^a^Chromosome. A and D represent A genome and D genome of cotton. ^b^(Major allele/minor allele). ^c^*a* and *d* represent the additive effect and the dominant effect respectively; *aa*, *ad*, *da* and *dd* denote the epistasis effects of the additive × additive, the additive × dominance, the dominance × additive, the dominance × dominance respectively; *de2* and *de*3 denote the interaction effects of the dominance with the second and the third environments respectively; *h*^2^ is the heritability in percentage due to genetic effect of significant QTS

A total of 21 significant QTSs were detected for the oil content, of which were 3 additive QTSs, 1 dominance QTS, 9 QTSs with both additive and dominance effects, and 4 paired epistatic QTSs, respectively (Table 2**,** Fig. 1 and Additional file 3: Table S3). Except QTS A9_85081637, whose additive heritability was almost twice as much as the dominance heritability, all the other QTSs activating in both additive and dominance exhibited lower additive heritability than their corresponding dominance heritability. Being the largest contributor to the total heritability and expressing the largest dominance heritability, QTS A13_83121382 not only exhibited additive, dominance and dominance-environment interaction effects, but also larger epistatic effects through interaction with A7_110987422 (*aa, ad, da, dd, ade2*). The total heritability of both A13_83121382 and A7_110987422, reached to 47.35%, accounting for over half of the total heritability of cottonseed oil content, indicating the importance of the two epistatic QTSs in regulating oil content. Both the phenotypic and the genotypic correlation analysis revealed significant negative correlation between the protein content and the oil content (*r*_*p*_ =  − 0.63, *r*_*g*_ =  − 0.92) (Additional file 4: Table S2). Association analysis detected a pleiotropic genetic variant D3_35705563 which contributed a positive additive effect to the protein content and positive additive and negative dominance effects to the oil content.

### Genetic architecture of the fatty acids

**Fig. 2:**
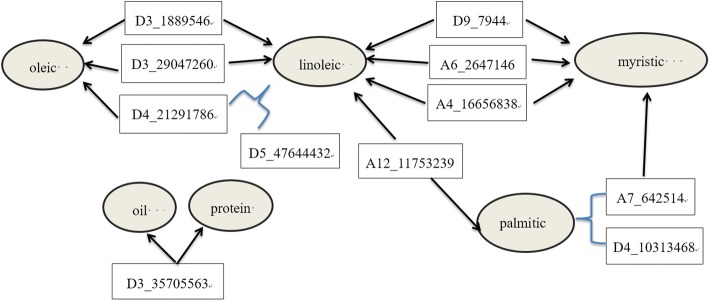
The genetic relationship among six cottonseed traits. The black arrow denotes individual genetic effect; blue brace denotes gene-gene epistatic interaction effect

Based on the above analysis on heritability, it could be concluded that the dominance effect played a core role in regulating the oleic acid. There were total 21 QTSs associated with the oleic acid (Table 3**,** Fig. 1 and Additional file 3: Table S3), consisting of 17 QTSs with additive or dominance effects, 2 QTSs with both additive effect and additive × environmental effect, 1 pair of QTSs with epistatic effect. Of 10 QTSs with dominance effects, 8 QTSs also contributed additive effects, and each dominance heritability was larger than the corresponding additive heritability except QTS A7_2266630. D3_1889546, contributing to the largest heritability, significantly affected the oleic acid by a positive dominance effect (${h}_d^2=20.28\%,d=1.019$). QTSs A1_44951529 and A1_85724143 was a unique pair of epistatic QTSs detected with a negative additive × additive effect (*aa* ≅  − 0.186) for the oleic acid, accounting for 2.69% epistatic heritability and 3.33% additive heritability. In the case of the linoleic acid, 21 QTSs were identified in total, including 19 main-effect QTSs and 1 pair of epistatic QTSs. For the oleic acid, the dominance effects predominated the variation, and were mainly attributed to the QTSs exhibiting multiple different effect types, the number of additive QTSs with a *p* value below 1 × 10^− 7^ was more than that of dominance effect QTSs. D4_21291786 and D5_47644432 not only contributed single locus effects, but also strong digenic additive × dominance and dominance × dominance effects which explained heritability of 24.68% for the linoleic acid. The oleic acid was negatively correlated with the linoleic acid in both phenotypic and genotypic effects (*r*_*p*_ =  − 0.54, *r*_*g*_ =  − 0.75) (Additional file 5: Table S2) and we detected 3 pleiotropic QTSs (D3_1889546, D3_29047260, D4_21291786) associated simultaneously with these two traits (Fig. 2**,** Additional file 6: Table S3). In addition to the negative specific additive effect in environment 1 (*ae*_*1*_) on the linoleic acid, D3_1889546 also exhibited a strong positive dominance effect on the oleic acid and a negative dominance effect on the linoleic acid with the largest dominance heritability 20.28 and 10.76%, respectively. D3_29047260 affected the oleic acid by the additive and environment-specific additive effects, but the linoleic acid by the dominance and environment-specific dominance effects. D4_21291786 was associated with the oleic acid through the significant additive and dominance effects, as well as with the linoleic acid by the significant additive effect and epistatic effects with the D5_47644432.

**Table 3:** Genome-wide significant QTSs associated with five fatty acids at -log_10_p > 7 (at least one kind of effect of QTS)

Trait	QTS	^a^Chr.	^b^Allele	^c^Effect type	Predict value	−log_10_*p*	*h*^*2*^(*%*)
Oleic	A1_44951529	A1	A/G	*a*	−0.276	17.8	2.98
	A1_85724143	A1	C/T	*a*	0.094	2.57	0.35
	A3_4717931	A3	C/T	*a*	0.139	3.99	0.76
		A3	C/T	*d*	−0.399	8.98	3.11
	A7_2266630	A7	A/G	*a*	0.288	18.18	3.24
		A7	A/G	*d*	0.357	2.27	2.48
	A12_120581335	A12	G/T	*a*	−0.275	14.45	2.95
		A12	G/T	*d*	0.452	9.43	3.99
	D1_1087912	D1	C/T	*d*	0.478	7.04	4.46
	D1_37367501	D1	A/G	*a*	0.171	7.29	1.14
	D3_1889546	D3	T/C	*d*	1.019	8.09	20.28
	D5_24437741	D5	A/G	*a*	0.263	15.41	2.70
		D5	A/G	*d*	−0.42	2.58	3.45
	D5_31125264	D5	G/A	*a*	−0.079	1.59	0.24
		D5	G/A	*d*	−0.4	8.31	3.11
	D9_148268	D9	T/C	*a*	−0.228	9.74	2.03
	D9_45944489	D9	T/C	*a*	0.17	7	1.13
	A1_44951529 & A1_85724143	A1 & A1	A/G & C/T	*aa*	−0.186	8.45	2.69
Linoleic	A6_69314946	A6	T/G	*a*	−0.268	8	1.10
	A9_23536520	A9	G/A	*a*	0.325	10.5	1.62
	A12_117532394	A12	G/A	*a*	−0.255	8.42	0.99
	A13_29883619	A13	T/C	*a*	0.388	16.99	2.30
		A13	T/C	*d*	−0.847	7.98	5.49
	D4_21291786	D4	G/A	*a*	−0.359	15.41	1.97
	D5_47644432	D5	G/C	*d*	0.47	3.67	1.69
	D6_59379832	D6	A/C	*a*	−0.275	9.34	1.15
		D6	A/C	*d*	0.853	3.63	5.56
	D4_21291786 & D5_47644432	D4 & D5	G/A & G/C	*aa*	−0.16	3.21	0.79
		D4 & D5	G/A & G/C	*ad*	0.505	3.95	3.89
		D4 & D5	G/A & G/C	*dd*	1.618	2.53	20.00
Palmitic	A1_61493378	A1	T/G	*a*	0.216	8.3	1.04
		A1	T/G	*d*	0.522	1.65	3.06
	A7_642514	A7	C/T	*a*	−0.572	51.11	7.36
	A11_600080	A11	A/G	*a*	0.293	12.82	1.93
	A12_117532394	A12	G/A	*a*	0.269	12.83	1.63
	A13_119809048	A13	G/A	*a*	0.258	11.54	1.50
		A13	G/A	*d*	1.025	6.27	11.82
	D4_10313468	D4	A/C	*a*	−0.254	10.73	1.45
		D4	A/C	*d*	−0.944	11.1	10.02
	D5_66975738	D5	A/G	*a*	−0.256	10.59	1.48
	D6_10836500	D6	T/C	*a*	−0.292	14.63	1.91
	A7_642514 & D4_10313468	A7 & D4	C/T & A/C	*aa*	−0.219	7.76	2.17
		A7 & D4	C/T & A/C	*da*	0.407	2.12	3.72
		A7 & D4	C/T & A/C	*dd*	1.809	5.89	36.79
Myristic	A1_35871478	A1	C/T	*a*	−0.014	10.62	2.54
		A1	C/T	*d*	−0.029	2.33	5.30
	A1_54364850	A1	T/C	*a*	0.017	15.02	3.52
	A1_67270927	A1	T/C	*a*	−0.014	8.52	2.34
		A1	T/C	*d*	−0.012	1.82	0.89
	A7_642514	A7	C/T	*a*	−0.02	18.61	4.70
		A7	C/T	*d*	−0.029	3.05	5.00
	A8_86207865	A8	G/A	*a*	−0.012	7.96	1.78
	A12_37853320	A12	G/A	*a*	0.006	2.03	0.46
		A12	G/A	*d*	0.026	7.3	4.13
	A13_19834182	A13	C/T	*a*	0.015	9.08	2.92
		A13	C/T	*d*	−0.026	10.99	4.25
Stearic	A4_17627308	A4	G/A	*a*	0.017	3.71	0.84
		A4	G/A	*ae1*	−0.045	8.27	6.13
		A4	G/A	*ae2*	−0.032	4.53	3.13
		A4	G/A	*ae3*	0.07	18.95	14.79
	A11_110829220	A11	A/G	*a*	0.025	7.46	1.83
	D1_10742184	D1	T/A	*a*	−0.024	7.15	1.74
	D10_30598593	D10	T/G	*ae1*	−0.019	1.51	1.04
		D10	T/G	*ae2*	−0.039	5.18	4.52
		D10	T/G	*ae3*	0.055	9.84	9.15
		D10	T/G	*de3*	0.035	1.54	1.89

^a^Chromosome. A represents A genome of cotton and D represents D genome of cotton. ^b^(Major allele/minor allele). ^c^*a*, *d*, *aa*, *ad*, *da* and *dd* denote the additive effect, the dominant effect, the epistasis effects of the additive × additive, the additive × dominance, the dominance × additive, the dominance × dominance respectively; *ae1*, *ae2* and *ae*3 denote the interaction effects of the additive with the first, the second and the third environments respectively, *de3* denotes the interaction effect of the dominance with the third environment. *h*^2^ is the heritability in percentage due to each genetic effect of significant QTSs

A total of 8 QTSs with a significant additive effect (−log_10_*p* > 8.30) were detected for the palmitic acid (Table 3**,** Fig. 1 and Additional file 3: Table S3). Three QTSs (A1_61493378, A13_119809048 and D4_10313468) showed the concordant effect direction, either positive dominance and additive effects or negative dominance and additive effects. The QTSs A7_642514 and D4_10313468 is a pair of key genetic variants, their total heritability reached 61.51% and explained 71.6% of the total heritability of the palmitic acid. They not only exhibited larger individual additive or dominance effects, as well as very strong epistatic effects, especially the dominance × dominance epistasis explaining as much as 36.79% of heritability.

On the genetic architecture of the myristic acid, 25 QTSs were identified, consisting of 12 QTSs with additive effect only, 10 QTSs with both additive and dominance effects, 1 QTS with additive and additive × environment effect and 1 pair of epistatic QTSs (Table 3**,** Fig. 1 and Additional file 3: Table S3). A1_35871478, expressing both additive and dominance effects, was the variant which accounted for the largest heritability by dominance effect, followed by A7_642514, whose dominance heritability was 5.00%. QTS pair A3_58421047 and A12_117532407 expressed a significant positive epistatic effect in environment 2 (*aae2*); A3_58421047 had no individual effect, whereas A12_117532407 contributed a positive additive effect, accounting for heritability of 1.47% (Additional file 3: Table S3). In addition to positive additive effect, A6_26471461 also had positive additive × environmental interaction effect in environment 2, and the interaction heritability (${h}_{ae}^2=1.07\%$) was over twice as that of its main effect (${h}_a^2=0.40\%$).

Different from the genetic basis of the fatty acid component traits discussed above, more genes of the stearic acid were found to be involved in environment interaction (Table 3**,** Fig. 1 and Additional file 3: Table S3) and no epistatic QTS was detected. Of 22 QTSs detected, 11 QTSs contributed additive or dominance × environment interaction effects. Apart from significant positive additive effect, A4_17627308 was also found to contribute the strongest additive × environment interaction effects in all 3 environments compared with other QTSs, its interaction heritability in environment 3 was larger than those in both environment 1 and environment 2, and the average gene-environment interaction heritability reached to 8%; furthermore, the effect direction of A4_17627308 in environment 3 was opposite to those in environment 1 and environment 2, implying that environment factor should be considered in the utilization of this genetic variant in breeding. QTS D10_30598593 was detected with not only significant additive × environment interaction effect in three environments but also dominance × environment interaction effect in environment 3.

According to the Fig. 2, we could find that fatty acids, except the stearic acid, were genetically linked mainly through pleiotropic QTSs and epistatic QTSs. There were in total 10 QTSs related to the genetic correlations of fatty acid composition, wherein, 8 QTSs were involved in the correlation of the linoleic acid with other 3 fatty acids (oleic, palmitic and myristic), indicating the key role of the linoleic acid in the genetic architecture of fatty acids. The A4_16656838 was a pleiotropic variant, which affected the myristic acid by significant additive effect, and the linoleic acid by significant additive and dominance effects. Similarly, the A6_26471461 and the D9_7944 were other two pleiotropic QTSs. The A6_26471461 affected the linoleic acid by significant negative additive effect and the additive × environment interaction effect in both environment 1 and environment 2, and the myristic acid by positive additive and positive additive-environment interaction effects in environment 2; while, the D9_7944 affected the linoleic acid and the myristic acid by opposite additive effects (*a* = 0.225 for the linoleic acid and *a* =  − 0.010 for the myristic acid). In addition, there seemed to exist an indirect genetic pathway connecting the linoleic acid and the myristic acid, in which A12_11753239 regulated the linoleic acid and the palmitic acid by opposite additive effects; while A7_642514 regulated the palmitic acid through individual negative additive effect and epistatic effect with D4_10313468, and also affected the myristic acid through negative additive and dominance effects.

### Candidate gene annotation

16 QTSs were identified in the regions of the annotated genes captured by 124 QTSs of cottonseed traits by using InterProScan (Table 4). A7_1504479, located in CDS of gene Gh_A07G0108, is associated with five kinds of protein domains relevant to crop resistance. Within these protein domains, protein kinase, catalytic domain (IPR000719), was found to participate in regulation on the lint yield of cotton in previous study [33]; Protein kinase-like domain (IPR011009) is related to the salt tolerance mechanism in *Arabidopsis thaliana* or rice [34, 35]; Kinase-like domain (IPR011009), which was found in cotton boll weevil transcriptome and associated with putative genes underling qNLB8.06_DK888_ of maize, is also relevant to insect control or disease resistance [36, 37]; Tyrosine-protein kinase, catalytic domain (IPR020635), relevant to A5_22579901, is associated with disease-related gene in rice [38]. In addition, A5_22579901, located in the CDS region of gene Gh_A05G1876, is associated with peptidase C48, SUMO/Sentrin/Ubl1 protein domain; D1_616439, located in 3’-UTR of gene Gh_D01G0083, encodes pseudouridine synthase family protein. A3_100487624, located in the CDS region of gene Gh_A10G1894, encoding SLC26A/SulP transporter (IPR001902), STAS domain (IPR002645), SLC26A/Sulphate transporter domain (IPR011547), SLC26A/SulP transporter (IPR001902), is associated with the oil content with concordant negative additive and dominance effects. However, A2_58832915 relevant to UDP-glusuronosyl/UDP-glucosyltransferase (IPR002213), and D1_62433793 relevant to light harvesting complex photosystem II, exhibited positive additive and dominance effects on the oil content, respectively.

**Table 4:** Candidate genes significantly associated with seven cottonseed traits

Trait	QTS	Candidate gene	Location	InterPro Description
Protein	A5_22579901	Gh_A05G1876	CDS	Peptidase C48, SUMO/Sentrin/Ubl1 (IPR003653)
	A7_1504479	Gh_A07G0108	CDS	Protein kinase domain (IPR000719); Serine-threonine/tyrosine-protein kinase, catalytic domain (IPR001245); Serine/threonine-protein kinase, active site (IPR008271); Protein kinase-like domain superfamily (IPR011009); Concanavalin A-like lectin/glucanase domain superfamily (IPR013320); Protein kinase, ATP binding site (IPR017441); Tyrosine-protein kinase, catalytic domain (IPR020635); Unknown (IPR002290)
	D1_616439	Gh_D01G0083	3’-UTR	Pseudouridine synthase, RsuA/RluB/C/D/E/F (IPR006145); Pseudouridine synthase, catalytic domain superfamily (IPR020103)
Oil	A2_58832915	Gh_D02G0225	CDS	UDP-glucuronosyl/UDP-glucosyltransferase (IPR002213)
	D1_62433793	Gh_D01G2232	5’-UTR	Chlorophyll A-B binding protein, plant (IPR001344); Chlorophyll A-B binding protein (IPR022796); Chlorophyll a/b binding domain superfamily (IPR023329);
	A3_100487624	Gh_A10G1894	CDS	SLC26A/SulP transporter (IPR001902); STAS domain (IPR002645); SLC26A/SulP transporter domain (IPR011547); Sulphate anion transporter, conserved site (IPR018045); Unknown (IPR030402)
Oleic	A7_2266630	Gh_D07G0133	intron	Armadillo-like helical (IPR011989); Uncharacterised domain NUC173 (IPR012978); Armadillo-type fold (IPR016024);
	D1_1087912	Gh_D01G0153	CDS	Pentatricopeptide repeat (IPR002885); Tetratricopeptide-like helical domain superfamily (IPR011990); DYW domain (IPR032867)
	A12_120581335	Gh_A12G2387	CDS	NA
Palmitic	D5_66975738	Gh_A04G0196	CDS	Transcription factor GRAS (IPR005202)
Palmitic,Myristic	A7_642514	Gh_A07G0057	CDS	Small GTPase superfamily (IPR001806); Ran GTPase (IPR002041); Small GTPase superfamily, Rho type (IPR003578); Unknown (IPR003579); Small GTP-binding protein domain (IPR005225); Small GTPase superfamily, Ras type (IPR020849); P-loop containing nucleoside triphosphate hydrolase (IPR027417)
Stearic	A2_25310067	Gh_A02G0110	CDS	Calreticulin/calnexin (IPR001580); Calreticulin/calnexin, P domain superfamily (IPR009033); Calreticulin (IPR009169); Concanavalin A-like lectin/glucanase domain superfamily (IPR013320); Calreticulin/calnexin, conserved site (IPR018124)
	A4_17627308	Gh_A03G0465	intron	Transferase (IPR003480); Chloramphenicol acetyltransferase-like domain superfamily (IPR023213);
	A11_32647777	Gh_D13G1997	up1k	MCM domain (IPR001208); DNA replication licensing factor Mcm (IPR008048); Nucleic acid-binding, OB-fold (IPR012340); Mini-chromosome maintenance, conserved site (IPR018525); P-loop containing nucleoside triphosphate hydrolase (IPR027417); MCM N-terminal domain (IPR027925); Mini-chromosome maintenance protein (IPR031327);
	A13_33729709	Gh_Sca004725G01	intron	Transferase (IPR003480); Chloramphenicol acetyltransferase-like domain superfamily (IPR023213);
	D1_10742184	Gh_A06G1283	up1k	Homeobox domain (IPR001356); START domain (IPR002913); Homeobox-like domain superfamily (IPR009057); START-like domain superfamily (IPR023393)

NA: not available

QTS A7_2266630, which was associated with the oleic acid and had larger additive heritability than the dominance heritability, is located in the intron of Gh_D07G0133, encoding Armadillo-like helical (IPR011989), Uncharacterised protein domain (IPR012978) and Armadillo-type fold (IPR016024). Another QTS, D1_1087912 that exhibited positive dominance effect (*d* = 0.478), is located in the CDS of Gh_D01G0153, which encodes Pentatricopeptide repeat (IPR002885), Tetratricopeptide-like helical domain superfamily (IPR011990) and DYW domain (IPR032867). D5_66975738 that was significantly associated with the palmitic acid by negative additive effect (*a* =  − 0.256, −log_10_*p* = 10.59)is located in the CDS of Gh_A04G0196. Pleiotropic A7_642514, affecting both the palmitic acid and the myristic acid, is located in the CDS of Gh_A07G0057, which encodes IPR001806, IPR002041, IPR003577, IPR003578, IPR003579, IPR005225, IPR020849 and IPR027417. For the stearic acid, A4_17627308 is located in the intron of Gh_A03G0465 and its annotated functions are transferase (IPR003480) and Chloramphenicol acetyltransferase-like domain superfamily (IPR023213); A2_25310067 is located in the CDS of Gh_A02G0110, whose function is Calreticulin/calnexin (IPR001580), Calreticulin/calnexin, P domain superfamily (IPR009033), Calreticulin (IPR009169), Concanavalin A-like lectin/glucanase domain superfamily (IPR013320), Calreticulin/calnexin, conserved site (IPR018124); in addition, there are other three candidate genes (Gh_D13G1997, Gh_Sca004725G01 and Gh_A06G1283) which are involved in the regulation on the stearic acid.

### Prediction of superior genotype for seven cottonseed traits

In order to assess the potential of these identified QTSs in genetic and molecular improvement of cottonseed traits, we conducted molecular design and genotypic value evaluation on the general superior homozygous line (GSL), the general superior hybrid line (GSH), the environment-specific superior homozygous line (SL) and the environment-specific superior hybrid line (SH), based on genetic effects of QTSs [39].

On the genetic architecture of seven cottonseed traits, genotypic values were consistently observed across three environments for the protein and the palmitic acid, because of no significant gene × environment interaction, whereas the genotypic values of other 5 traits varied (Additional file 5: Table S4). Obviously, both the pure line (homozygous genotype: QQ, qq, and SL) and the hybrid line (SH) possessed potential to improve 7 cottonseed traits, and hybrid line exhibited more advantages than the pure line in terms of the range of genetic values achieved in designed lines. The oleic acid and the myristic acid had the highest SH and the highest SL value in environment 1 or in environment 2 respectively. Analogously, the linoleic acid and the stearic acid, influenced by both the additive × environment and the dominance × environment interaction effects, could achieve the highest SH and SL values in environment 2 or in environment 3. Different from other traits influenced by gene × environment interaction, the genotypic values of both SL and SH (SL(+) and SH(+)) remained constant across 3 environments for the oil content.

Because there was no significant gene × environment interaction for the protein content and the palmitic acid, the designed superior genotypes (GSL(+), GSH(+)) based on genetic main effects of QTSs would keep unchanged in every environment (Table 5 and Additional file 6: Table S5). Notably, although the oil content, the oleic acid, and the myristic acid were influenced by gene × environment interaction, the same superior homogenous genotypes for all three environments could be designed, as well as superior hybrid genotypes, for each trait, respectively. To design QTS genotype for synchronous improvement of multiple cottonseed traits, it is evaluable to classify all other detected QTSs into two groups, one consists of non-pleiotropic QTS and the other consists of pleiotropic QTS. The designed genotypes for each QTS residing in the regions of annotated genes were presented in the upper part above the middle split line of the Table 5 and the lower part for the pleiotropic QTSs. The Table 5 showed that selecting AA at the A7_1504479, TT at the D1_616439, and AA at the A5_22579901 could achieve the maximum genetic value of the protein content by the superior homozygous lines; similarly, GG at the A2_58832915, and AA at the A3_100487624 would be preferred for homozygous lines for the oil content, GG at the D5_66975738 for the palmitic acid, AA at the A13_128192242 for the myristic acid; however, GG and AG at the D1_62433793 were optimal for the superior homozygous lines and the superior hybrids for the oil content, respectively, and similar designs could be found at A7_2266630, A12_120581335, and D1_1087912 for the oleic acid.

**Table 5:** The genotypes achieving the maximum genetic value in designed lines at the QTSs in annotated genes and the QTSs with pleiotropic effects

QTS	Trait	GSL(+)	SL(+)1	SL(+)2	SL(+)3	GSH(+)	SH(+)1	SH(+)2	SH(+)3
A7_1504479	Protein	AA	AA	AA	AA	AA	AA	AA	AA
D1_616439	Protein	TT	TT	TT	TT	TT	TT	TT	TT
A5_22579901	Protein	AA	AA	AA	AA	AA	AA	AA	AA
A7_642514	Palmitic	TT	TT	TT	TT	TT	TT	TT	TT
D5_66975738	Palmitic	GG	GG	GG	GG	GG	GG	GG	GG
A2_58832915	Oil	GG	GG	GG	GG	GG	GG	GG	GG
A3_100487624	Oil	AA	AA	AA	AA	AA	AA	AA	AA
D1_62433793	Oil	GG	GG	GG	GG	AG	AG	AG	AG
A7_2266630	Oleic	AA	AA	AA	AA	AG	AG	AG	AG
A12_120581335	Oleic	TT	TT	TT	TT	GT	GT	GT	GT
D1_1087912	Oleic	CC	CC	CC	CC	CT	CT	CT	CT
A7_642514	Myristic	TT	TT	TT	TT	TT	TT	TT	TT
A13_128192242	Myristic	AA	AA	AA	AA	AA	AA	AA	AA
A2_25310067	Stearic	CC	CC	CC	CC	CC	CC	CT	CC
A4_17627308	Stearic	GG	AA	AA	GG	GG	AA	AA	GG
A11_32647777	Stearic	GG	GG	GG	GG	GG	GG	GG	GG
A13_33729709	Stearic	TT	CC	TT	TT	TC	TC	TC	TC
D1_10742184	Stearic	AA	AA	AA	AA	AA	AA	AA	AA
D3_35705563	Oil	TT	TT	TT	TT	TT	TT	TT	TT
D3_35705563	Protein	CC	CC	CC	CC	CC	CC	CC	CC
A4_16656838	Myristic	GG	GG	GG	GG	GG	GG	GG	GG
A4_16656838	Linoleic	AA	AA	AA	AA	AA	AA	AA	AA
A6_26471461	Myristic	AA	AA	AA	AA	AA	AA	AA	AA
A6_26471461	Linoleic	GG	GG	GG	GG	GG	GG	GG	GG
A12_117532394	Palmitic	GG	GG	GG	GG	GG	GG	GG	GG
A12_117532394	Linoleic	AA	AA	AA	AA	AA	AA	AA	AA
D9_7944	Myristic	TT	TT	TT	TT	TT	TT	TT	TT
D9_7944	Linoleic	CC	CC	CC	CC	CC	CC	CC	CC
D3_29047260	Oleic	TT	TT	TT	TT	TT	TT	TT	TT
D3_29047260	Linoleic	TT	TT	TT	TT	TT	TT	TT	TT
A7_642514	Palmitic	TT	TT	TT	TT	TT	TT	TT	TT
A7_642514	Myristic	TT	TT	TT	TT	TT	TT	TT	TT
D4_10313468	Palmitic	CC	CC	CC	CC	CC	CC	CC	CC
D4_21291786	Oleic	GG	GG	GG	GG	GG	GG	GG	GG
D4_21291786	Linoleic	AA	AA	AA	AA	AA	AA	AA	AA
D5_47644432	Linoleic	GG	GG	GG	GG	GG	GG	GG	GG
D3_1889546	Oleic	TT	TT	TT	TT	TC	TC	TC	TC
D3_1889546	Linoleic	TT	CC	TT	TT	TT	CC	TT	TT

GSL and GSH stand for the superior homozygous line and the superior hybrid without consideration of gene by environment interaction respectively; SL and SH stand for the environment-specific superior homozygous line and the environment-specific superior hybrid with consideration of gene by environment interaction respectively; the sign “+” in parentheses denotes the line could achieve the maximum genetic value in all designed genotypes; the number 1,2, and 3 at the right of the parentheses are environment codes; the upper part above the middle line of the table is for the QTSs residing in annotated genes; the lower part is for the QTSs exhibiting pleiotropic effects

On the linoleic acid and the stearic acid, the designed genotypes at most QTSs exhibited greater consistency across different environments except QTS D3_1889546 for the linoleic acid and 7 QTSs (A2_25310067, A4_135747886, A4_17627308, A12_78651650, D5_44746794, D10_5643096, D10_30598593) (Table 4, Additional file 6: Table S5) for the stearic acid. On the stearic acid, the design of superior genotype was more complex due to gene-environment interaction. For example, CC at A2_25310067 was preferred for achieving the maximum genetic value of the stearic acid for the superior homozygous lines (GSL, SL) and the superior hybrids (GSH, SH) in environment 1 and 3, whereas CT was better in environment 2 for the superior hybrids.

Pleiotropic genes make it more difficult to conduct synchronous improvement of multiple crop traits. Out of 11 pleiotropic QTSs (Table 5), the genotypes of 5 QTSs (D3_35705563, A4_16656838, A6_26471461, A12_117532394 and D9_7944), exhibited identical across superior lines (GSL, SL, GSH, SH) for same trait, but completely different between correlated traits. For example, CC at D3_35705563 was preferred for achieving the maximum genetic value of the protein content, but completely different genotype TT for the oil content, which was in concert with the negative genetic correlation between two traits. Selecting TT at D3_29047260 was a better choice to improve the linoleic acid, and the oleic acid simultaneously. On selection of epistatic effect between A7_642514 and D4_10313468, TT at A7_642514 together with CC at D4_10313468 was preferred for both the palmitic acid and the myristic acid; likewise, selecting GG at D5_47644432 together with GG or AA at D4_21291786 for the oleic acid and for the linoleic acid, respectively, was preferred. TT in superior homogenous design (GSL, SL) and TC in superior hybrid design (GSH, SH) were preferred in all environments for the oleic acid.

Myristic acid and palmitic acid could increase the level of low-density lipoprotein (LDL) cholesterol in serum, it is desired to reduce the components of myristic acid and palmitic acid in cottonseed for human health; thus, the optimal genotypes are expected to lower myristic acid and palmitic acid of cottonseed, which are provided in the Additional file 7**:** Table S6. Such information on designed genotypes will bring new insight into the molecular improvement of cottonseed traits.

## Discussion

Mixed linear model approach has been successfully applied in linkage mapping of complex traits, including seed traits in crops [32, 40, 41]. Because of the advantages of the mixed linear model approach in aggregating total genetic effects of numerous genetic variants scattered in the whole genome and dealing with covariates, such as age, sex and population stratification, many tools have been developed based on mixed linear model for estimating total genetic heritability and performing genome wide association mapping [42–45]. However, genetic effects due to epistasis or gene × environment interaction (interactions of one gene or two paired genes with environment) are ignored in most of these methods, such as the method proposed by Lü et al. [45], whose model ignored the interaction of epistasis with environment [42–45]. More recently, the method proposed by Zhang et al. [46] included effects of additive, dominance, epistasis and their interaction effects with environment in a mixed linear model to conduct genome wide association mapping and the corresponding software QTXNetowork has been available (http://ibi.zju.edu.cn/software/). Thus, our study employed this new method and software to explore intricate genetic architecture of cottonseed traits. Although relatively small compared with the main effects of an individual locus (e.g., additive and dominance) for most of the cottonseed traits studied, significant epistatic effects and gene-environment interactions were detected, justifying the effectiveness of the method we employed.

It should be noted that we first performed filtering on total ~ 390 K SNPs using the generalized multifactor dimensionality reduction (GMDR) [47, 48], and then the filtered SNPs were used in association mapping by the QTXNetwork. The effectiveness of this strategy has been simulated and confirmed recently [49]. Considering the influence of potential population stratification on association mapping, we conducted population stratification analysis based on 203,021 unlinked SNPs and two subpopulations were found in the 316 accessions (Additional file 1**:** Figure S2). Under the assumption of two subpopulations existent in our cotton population, chi-square test was applied to investigate significance of difference in genotypic frequency between subpopulations at the QTSs detected by our strategy. It was observed that the *p*-values of 120 of 124 QTSs were not significant after Bonferroni adjustment (*p*-value < 2.46 × 10^− 7^); However, 4 QTSs (A11_110829220, A13_21415280, D10_30598593 and D10_5643096) had significant difference in frequency between subpopulations.

It has been well documented that epistasis and gene by environment interaction are mostly involved in the genetic architecture of most agronomic, yield and quality traits in crops. Liu et al. reported that 12 QTLs were detected for the protein content using IF_2_ population, of which 6 QTLs interacted with environment [7]. Zeng et al. (2016) investigated the association of SNPs in *GhSus* family genes with fiber- and seed-related traits in 277 upland cotton accessions by epistatic association mapping (EAM) model. For cottonseed-related traits, one main-effect quantitative trait nucleotides (QTNs) and one epistatic QTN were found to be associated with seed oil content and protein content respectively, but no significant one-dimensional gene by environment interaction was found [29]. In our study, in addition to additive or dominance effect, we also detected significant epistatic effects on the oil content and the protein content; however, significant epistasis × environment interaction effect was detected only on the oil content and not on the protein content. These results may be due to the difference in environmental factors and genetic materials employed in these studies.

Bioinformatics analysis showed that the QTS A7_1504479, is located in the CDS of gene Gh_A07G0108, and associated with protein domains relevant to resistance mechanism in rice, maize, etc.; thus, further study on Gh_A07G0108 may reveal more information about resistance mechanism of cotton. Many studies have detected negative correlation between the protein content and the oil content in cottonseed and other oil crops, such as in soybean and sesame [8, 12, 14, 50]. Our study detected the key QTS D3_35705563 controlling these two traits by additive and dominance effects simultaneously. Both the oleic acid and the linoleic acid have medical efficacy in lowering LDL-cholesterol, thus they could reduce one’s risk of cardiovascular disease. Oleic acid could be converted into linoleic acid by the delta-12 fatty acid desaturase gene [51, 52], this genetic relationship might be related to 3 significant pleiotropic QTSs (D3_1889546, D3_29047260, D4_21291786) which affect both traits by additive effect, dominance effect, epistatic effect, as well as gene × environmental effect.

Currently, some studies have reported that variation of complex traits result from non-coding regulatory variants more frequently than from coding sequence polymorphisms [53]. Similarly, our study also found that most QTSs accounting for significant heritability are located in non-coding region and play a key role in dissection of the genetic regulation mechanism and the relationship among traits. Further studies, including the functional verification of annotated genes through molecular experiments and the QTL-GWAS joint analysis will be necessary for us to identify the real causal variants.

## Conclusions

In summary, this study has detected significant epistasis and gene by environment interactions in the genetic architecture of seven cottonseed traits using association analysis based on a QTS full model and high density SNPs in cotton. The results on the genetic information of the distinguished variants and the superior genotype design provide new vision on the molecular mechanism and insight into marker-assisted selection (MAS) strategy on efficient molecular improvement of the traits in cotton.

## Methods

### Plant materials and phenotyping

316 tetraploid cotton accessions were selected according to the diversity in geographical origins, phenotypic variation, which were provided by the National Cotton Mid-term GeneBank (Anyang, Henan, China). In 2009, all accessions were planted with three replicates in 3 regions of three China provinces (E1 = Anyang of Henan (114.35^。^E,36.10^。^N), E2 = Kuche of Xinjiang (82.97^。^E,41.68^。^N), E3 = Nanjing of Jiangsu (118.46^。^E,32.03^。^N). The seeds of each replicate were collected and the protein, the oil (total of all fatty acid) and its components including oleic acid, linoleic acid, palmitic acid, myristic acid and stearic acid were tested three times each replicate. The names of all 316 accessions and the summary statistics of seven seed traits were presented in the supplement file (Additional file 8: Fig. S1, Additional file 9: Table S1).

The seed kernel proteins of 316 accessions were determined by Kjeldahl method referred to National food safety standard determination of protein in foods (GB 5009.5–2010). The seed kernel oil was measured by reference to animal and vegetable fats and oils--determination of moisture and volatile matter content (GB/T 9696–2008/ISO662:1998). The contents of seed kernel fatty acids including almitic, linoleic, oleic, myristic, stearic etc. in each accessions were assayed by gas chromatography (GC) using a 7890A chromatograph (Agilent tech., Wilmington, USA) according to the national standard for animal and vegetable fats and oils analysis by gas chromatography of methyl esters of fatty acids (GN/T17377–2008/ISO5508:1990, IDT) and national standard for fatty acid test in the food (GB 5009.168–2016). This measurement was carried out by the Laboratory of Quality & Safty Risk Assessment for Oilseed Products (Wuhan), MOA, PR China. Since the absolute concentration is not the crucial factor for the fatty acids in 316 accession population, the internal normalization method was employed as a quantitative tool for relative amounts estimation of fatty acids analysis in this research.

The peaks of different fatty acids were estimated by the reference for retention time and relative retention time of certain fatty acids. The reference for retention time (min) of the myristic acid, the palmitic acid, the stearic acid, the oleic acid and the linoleic acid, are 32.45, 38.81, 42.27, 44.38, and 47.73, respectively. The calculation for fatty acid components as follows, and the absolute difference of two experiment replications is less than 10% of arithmetic mean value,

$$ {X}_i=\raisebox{1ex}{${A}_i\times 100$}\!\left/ \!\raisebox{-1ex}{$\sum A$}\right. $$

where *X*_*i*_ is the content of a certain ingredient (%), *A*_*i*_ is the peak area of a certain ingredient, ∑*A* is the total area of all ingredients.

### Genotyping by sequencing

Total genomic DNA was extracted from the leaf tissues of 316 accessions using CTAB method with some modifications; the sequencing on all the accessions was conducted in 2013. The paired-end reads were mapped to the reference genome of *G. arboreum* (v1.0, http://cgp.genomics.org.cn/) and G. *raimondii* (v2.0, http://www.phytozome.net/) by the software BWAv0.5.927. SNP calling procedure was carried out with realSFS (v0.983, http://128.32.118.212/thorfinn/realSFS/) based on the Bayesian estimation. SNPs matching the following criteria were removed for quality control: (1) sequencing depth is greater than 6500× or less than 60×; (2) the distance between two adjacent SNP loci is shorter than 5 bp; (3) the call rate is less than 70% in the whole population; (4) the proportion of its heterozygous genotypes is more than 30%; and (5) minor allele frequency (MAF) is less than 0.01. Eventually, a total of 390,612 SNPs were obtained for association analysis.

### Population structure analysis

The software PLINK [25] was applied in calculation of LD measured by the average pairwise correlation coefficient (*r*^2^), and selection of unlinked SNPs. The possible population structure was analyzed by the fastStructure software [54]. After excluding SNPs that have a *r*^2^ value larger than 0.1 with any other SNP within a 50-SNP sliding window, a total of 203,021 SNPs were retained. The hypothetical number of subpopulations (*K*) from 1 to 10 was performed at five independent run iterations using 203,021 unlinked SNPs by the fastStructure. The output of the fastStructure was used to estimate *K* according to the method described by Raj et al. [54].

### Association mapping

Mixed linear approach was used in the GWAS on seven cottonseed traits of 316 accessions. The statistical model consists of the effects of environment, additive, dominance, additive × additive, additive × dominance, dominance × additive, and dominance × dominance epistatic effects, as well as their interactions with environments. The genetic effects were treated as fixed effects, and the environment effects and gene-environment interactions as random effects. Suppose one surveyed trait is controlled by *s* loci (QTSs), then, the phenotype *y*_*kh*_ of the *k*-th genotype in the *h*-th environment can be expressed by following mixed linear model:

$$ {\displaystyle \begin{array}{c}{y}_{kh}=\mu +\sum \limits_i{a}_i{x}_{A_{ik}}+\sum \limits_i{d}_i{x}_{D_{ik}}+\sum \limits_{i<j}{aa}_{ij}{x}_{A{A}_{ij k}}+\sum \limits_{i<j}{ad}_{ij}{x}_{A{D}_{ij k}}+\sum \limits_{i<j}{da}_{ij}{x}_{D{A}_{ij k}}+\sum \limits_{i<j}{dd}_{ij}{x}_{D{D}_{ij k}}\\ {}+{e}_h+\sum \limits_i{ae}_{ih}{u}_{A_{ik}{E}_h}+\sum \limits_i{de}_{ih}{u}_{D_{ik} Eh}+\sum \limits_{i<j}{aa e}_{ij h}{u}_{A{A}_{ij k}{E}_h}+\sum \limits_{i<j}{ad e}_{ij h}{u}_{A{D}_{ij k}{E}_h}\\ {}+\sum \limits_{i<j}{da e}_{ij h}{u}_{D{A}_{ij k}{E}_h}+\sum \limits_{i<j}{dd e}_{ij h}{u}_{D{D}_{ij k}{E}_h}+{\varepsilon}_{kh}\end{array}} $$

Where *μ* is the population mean; *a*_*i*_ and *d*_*i*_ are the additive and the dominance effects of the *i*-th QTS, with coefficients ${x}_{A_{ik}}\left(=1,0,-1\right)$ and ${x}_{D_{ik}}\left(=0,1,0\right)$ for the QTS genotype *QQ*, *Qq* and *qq*, respectively; *aa*_*ij*_, *ad*_*ij*_, *da*_*ij*_ and *dd*_*ij*_ are the additive-additive, the additive-dominance, the dominance-additive, the dominance-dominance epistatic effects between the *i*-th QTS and the *j*-th QTS, with coefficients ${x}_{AA_{ijk}}={x}_{A_{ik}}\cdot {x}_{A_{jk}}$, ${x}_{AD_{ijk}}={x}_{A_{ik}}\cdot {x}_{D_{jk}}$, and ${x}_{DD_{ijk}}={x}_{D_{ik}}\cdot {x}_{D_{jk}}$, respectively; ${e}_h\sim \left(0,{\sigma}_E^2\right)$ is the random effect of the *h*-th environment; ${ae}_{ih}\sim \left(0,{\sigma}_{A_iE}^2\right)$ and ${de}_{ih}\sim \left(0,{\sigma}_{D_iE}^2\right)$ are the interaction effects of the additive, the dominance of the *i*-th QTS and the *h*-th environment, random, with coefficients ${u}_{A_{ik}{E}_h}\left(={x}_{A_{ik}}\right)$ and ${u}_{D_{ik}{E}_h}\left(={x}_{D_{ik}}\right)$, respectively; ${aae}_{ij h}\sim \left(0,{\sigma}_{AA_{ij}E}^2\right)$, ${ade}_{ij h}\sim \left(0,{\sigma}_{AD_{ij}E}^2\right)$, ${dae}_{ij h}\sim \left(0,{\sigma}_{DA_{ij}E}^2\right)$ and ${dde}_{ij h}\sim \left(0,{\sigma}_{DD_{ij}E}^2\right)$ are the interaction effects of four epistasis components of the *i*-th QTS and *j*-th QTS with *h*-th environment, random, with coefficients ${u}_{AA_{ijk}{E}_h}\left(={x}_{AA_{ijk}}\right)$, ${u}_{AD_{ijk}{E}_h}\left(={x}_{AD_{ijk}}\right)$, ${U}_{DA_{ijk}{E}_h}\left(={x}_{DA_{ijk}}\right)$ and ${u}_{DD_{ijk}{E}_h}\left(={x}_{DD_{ijk}}\right)$, respectively; ${\varepsilon}_{kh}\sim \left(0,{\sigma}_{\varepsilon}^2\right)$ is the residual effect, random.

The software QTXNetwork, developed for the mixed linear model-based association analysis method by Zhang et al. [46], was employed to analyze the above model. Since the number and the positions of all significant SNPs involved in variation of the surveyed trait are unknown, the QTXNetwork first use 1-dimensional and 2-dimensional genome scanning to distinguish all candidate SNPs with significant single-locus effects and all paired SNPs with significant epistatic effects; finally, a full QTS model was established to estimate all quantitative trait SNP (QTS) parameters. The detail of this mapping strategy can refer to the paper by Zhang et al. [46].

In order to alleviate enormous computational burden and exponentially increased multiple testing for detecting epistasis, we used generalized multifactor dimensionality reduction method (GMDR) [47] and the corresponding software GMDR-GPU to screen potential trait-associated SNPs [48]; In order to screen SNPs potentially associated with the traits as many as possible, two different scanning strategies for GMDR analysis were separately conducted, at the level of three dimensional interaction, for each traits; one is ignoring environmental factor and the other is including environmental factor as a covariate in the GMDR model. Then, all screened SNPs by two strategies were used in association analysis by the QTXNetwork software [46]. In order to control the experiment-wise type I error rate, 1000 permutation testing for each SNP and each pair of SNPs were employed to determine the empirical threshold value of the F-statistic at the experiment significance level of 0.05. Finally, a full QTS model was established by all significant QTSs and the genetic effects of QTSs were predicted using a Monte Carlo Markov Chain method with 20,000 Gibbs sampler iterations.

## Additional files

Additional file 1: **Figure S2.** Population structure of 316 cotton accessions based on pruned unlinked SNPs. (DOC 69 kb)

Additional file 2: **Figure S3.** Genome-wide average LD decay estimated based on the population of 316 cotton accessions. (DOC 58 kb)

Additional file 3: **Table S3.** The other genome-wide significant QTSs associated with five fatty acids. (DOC 185 kb)

Additional file 4: **Table S2.** Phenotypic and genotypic correlation between seven cottonseed traits. (DOC 58 kb)

Additional file 5: **Table S4.** Predicted genetic values (G) for QQ, qq, Qq, superior homozygous line (SL), and superior hybrids (SH) of seven cottonseed traits. (DOC 114 kb)

Additional file 6: **Table S5.** The SNP genotypes achieving the maximum genetic value of seed traits in designed superior homozygous lines and hybrids at the QTSs absent in the Table 5. (DOC 168 kb)

Additional file 7: **Table S6.** The SNP genotypes achieving the minimum genetic value of seed traits in designed lines for QTSs (DOC 210 kb)

Additional file 8: **Figure S1.** The observed phenotype distribution of seven traits. (DOC 83 kb)

Additional file 9: **Table S1.** The information of 316 accessions and the summary statistics of seven seed traits (DOC 321 kb)

## Abbreviation

EAM: Epistatic association mapping
GMDR: Generalized multifactor dimensionality reduction
GWAS: Genome-wide association study
MAS: Marker-assisted selection.
QTL: Quantitative trait loci
QTN: Quantitative trait nucleotides
QTS: Quantitative trait SNP

## References

[1] Davis LC. Modification of oil content in cottonseed using chemical mutagenesis: Texas Tech University; 2015.

[2] Li MH, Robinson EH (2006). Use of cottonseed meal in aquatic animal diets: a review. N Am J Aquac.

[3] Wang X, Zhang H, Wu S, Yue H, Wang J, Li J, Qi G (2015). Dietary protein sources affect internal quality of raw and cooked Shell eggs under refrigerated conditions. Asian Australas J Anim Sci.

[4] O’Brien R, Wakelyn P. Cottonseed oil: an oil for trans-free options. Inform. 2005;16(11). http://www.ncga.cotton.org/tech/cottonseed/upload/05CottonseedOil.Revised.pdf.

[5] Rashid U, Anwar F, Knothe G (2009). Evaluation of biodiesel obtained from cottonseed oil. Fuel Process Technol.

[6] Alfred Q, Liu HY, Xu HM, Li JR, Wu JG, Zhu SJ, Shi CH (2012). Mapping of quantitative trait loci for oil content in cottonseed kernel. J Genet.

[7] Liu H, Quampah A, Chen J, Li J, Huang Z, He Q, Shi C, Zhu S (2012). QTL analysis for gossypol and protein contents in upland cottonseeds with two different genetic systems across environments. Euphytica.

[8] Yu J, Yu S, Fan S, Song M, Zhai H, Li X, Zhang J (2012). Mapping quantitative trait loci for cottonseed oil, protein and gossypol content in a Gossypium hirsutum× Gossypium barbadense backcross inbred line population. Euphytica.

[9] Shang L, Abduweli A, Wang Y, Hua J (2016). Genetic analysis and QTL mapping of oil content and seed index using two recombinant inbred lines and two backcross populations in upland cotton. Plant Breed.

[10] Liu H, Quampah A, Chen J, Li J, Huang Z, He Q, Shi C, Zhu S (2017). QTL mapping with different genetic systems for nine non-essential amino acids of cottonseeds. Mol Gen Genomics.

[11] Huang X, Han B (2014). Natural variations and genome-wide association studies in crop plants. Annu Rev Plant Biol.

[12] Hwang E-Y, Song Q, Jia G, Specht JE, Hyten DL, Costa J, Cregan PB (2014). A genome-wide association study of seed protein and oil content in soybean. BMC Genomics.

[13] Wei X, Liu K, Zhang Y, Feng Q, Wang L, Zhao Y, Li D, Zhao Q, Zhu X, Zhu X (2015). Genetic discovery for oil production and quality in sesame. Nat Commun.

[14] Badigannavar A, Myers GO (2015). Genetic diversity, population structure and marker trait associations for seed quality traits in cotton (Gossypium hirsutum). J Genet.

[15] Lee S-B, Kaittanis C, Jansen RK, Hostetler JB, Tallon LJ, Town CD, Daniell H (2006). The complete chloroplast genome sequence of Gossypium hirsutum: organization and phylogenetic relationships to other angiosperms. BMC Genomics.

[16] Wang K, Wang Z, Li F, Ye W, Wang J, Song G, Yue Z, Cong L, Shang H, Zhu S (2012). The draft genome of a diploid cotton Gossypium raimondii. Nat Genet.

[17] Zhu YX, Li FG (2013). The Gossypium raimondii genome, a huge leap forward in cotton genomics. J Integr Plant Biol.

[18] Li F, Fan G, Wang K, Sun F, Yuan Y, Song G, Li Q, Ma Z, Lu C, Zou C (2014). Genome sequence of the cultivated cotton Gossypium arboreum. Nat Genet.

[19] Li F, Fan G, Lu C, Xiao G, Zou C, Kohel RJ, Ma Z, Shang H, Ma X, Wu J (2015). Genome sequence of cultivated upland cotton (Gossypium *hirsutum* TM-1) provides insights into genome evolution. Nat Biotechnol.

[20] Zhang T, Hu Y, Jiang W, Fang L, Guan X, Chen J, Zhang J, Saski CA, Scheffler BE, Stelly DM (2015). Sequencing of allotetraploid cotton (Gossypium *hirsutum* L. acc. TM-1) provides a resource for fiber improvement. Nat Biotechnol.

[21] Byers RL, Harker DB, Yourstone SM, Maughan PJ, Udall JA (2012). Development and mapping of SNP assays in allotetraploid cotton. Theor Appl Genet.

[22] Hulse-Kemp AM, Lemm J, Plieske J, Ashrafi H, Buyyarapu R, Fang DD, Frelichowski J, Giband M, Hague S, Hinze LL (2015). Development of a 63K SNP Array for Cotton and High-Density Mapping of Intra-and Inter-Specific Populations of Gossypium spp. G3: Genes| Genomes| Genetics.

[23] Yang J, Hu C, Hu H, Yu R, Xia Z, Ye X, Zhu J (2008). QTLNetwork: mapping and visualizing genetic architecture of complex traits in experimental populations. Bioinformatics.

[24] Bradbury PJ, Zhang Z, Kroon DE, Casstevens TM, Ramdoss Y, Buckler ES (2007). TASSEL: software for association mapping of complex traits in diverse samples. Bioinformatics.

[25] Purcell S, Neale B, Todd-Brown K, Thomas L, Ferreira MA, Bender D, Maller J, Sklar P, De Bakker PI, Daly MJ (2007). PLINK: a tool set for whole-genome association and population-based linkage analyses. Am J Hum Genet.

[26] Beaty TH, Ruczinski I, Murray JC, Marazita ML, Munger RG, Hetmanski JB, Murray T, Redett RJ, Fallin MD, Liang KY (2011). Evidence for gene-environment interaction in a genome wide study of nonsyndromic cleft palate. Genet Epidemiol.

[27] Liu Y, Maxwell S, Feng T, Zhu X, Elston RC, Koyutürk M, Chance MR (2012). Gene, pathway and network frameworks to identify epistatic interactions of single nucleotide polymorphisms derived from GWAS data. BMC Syst Biol.

[28] Xu S (2013). Mapping quantitative trait loci by controlling polygenic background effects. Genetics.

[29] Zeng Y-D, Sun J-L, Bu S-H, Deng K-S, Tao T, Zhang Y-M, Zhang T-Z, Du X-M, Zhou B-L (2016). EcoTILLING revealed SNPs in GhSus genes that are associated with fiber-and seed-related traits in upland cotton. Sci Rep.

[30] Huang X, Wei X, Sang T, Zhao Q, Feng Q, Zhao Y, Li C, Zhu C, Lu T, Zhang Z (2010). Genome-wide association studies of 14 agronomic traits in rice landraces. Nat Genet.

[31] Riedelsheimer C, Czedik-Eysenberg A, Grieder C, Lisec J, Technow F, Sulpice R, Altmann T, Stitt M, Willmitzer L, Melchinger AE (2012). Genomic and metabolic prediction of complex heterotic traits in hybrid maize. Nat Genet.

[32] Abdurakhmonov I, Kohel R, Yu J, Pepper A, Abdullaev A, Kushanov F, Salakhutdinov I, Buriev Z, Saha S, Scheffler B (2008). Molecular diversity and association mapping of fiber quality traits in exotic G. *hirsutum* L. germplasm. Genomics.

[33] Jia Y, Sun X, Sun J, Pan Z, Wang X, He S, Xiao S, Shi W, Zhou Z, Pang B (2014). Association mapping for epistasis and environmental interaction of yield traits in 323 cotton cultivars under 9 different environments. PLoS One.

[34] Guo M, Yang A, Zhou C, Liu X (2012). The new understanding of Arabidopsis thaliana proteins associated with salinity. J Plant Interact.

[35] Wang J, Chen L, Wang Y, Zhang J, Liang Y, Xu D (2013). A computational systems biology study for understanding salt tolerance mechanism in rice. PLoS One.

[36] Chung C-L, Jamann T, Longfellow J, Nelson R (2010). Characterization and fine-mapping of a resistance locus for northern leaf blight in maize bin 8.06. Theor Appl Genet.

[37] Firmino AAP, de Assis Fonseca FC, de Macedo LLP, Coelho RR, de Souza JDA, Togawa RC, Silva-Junior OB, Pappas-Jr GJ, da Silva MCM, Engler G (2013). Transcriptome analysis in cotton boll weevil (Anthonomus grandis) and RNA interference in insect pests. PLoS One.

[38] Zhang Q-J, Zhu T, Xia E-H, Shi C, Liu Y-L, Zhang Y, Liu Y, Jiang W-K, Zhao Y-J, Mao S-Y (2014). Rapid diversification of five Oryza AA genomes associated with rice adaptation. Proc Natl Acad Sci.

[39] Yang J, Zhu J (2005). Methods for predicting superior genotypes under multiple environments based on QTL effects. Theor Appl Genet.

[40] Qi T, Cao YJ, Cao LY, Gao YM, Zhu SJ, Lou XY, Xu HM (2015). Dissecting genetic architecture underlying seed traits in multiple environments. Genetics.

[41] Qi T, Jiang B, Zhu Z, Wei C, Gao Y, Zhu S, Xu H, Lou X (2014). Mixed linear model approach for mapping quantitative trait loci underlying crop seed traits. Heredity.

[42] Yang JA, Benyamin B, McEvoy BP, Gordon S, Henders AK, Nyholt DR, Madden PA, Heath AC, Martin NG, Montgomery GW (2010). Common SNPs explain a large proportion of the heritability for human height. Nat Genet.

[43] Yang JA, Lee SH, Goddard ME, Visscher PM (2011). GCTA: a tool for genome-wide complex trait analysis. Am J Hum Genet.

[44] Zhang ZW, Ersoz E, Lai CQ, Todhunter RJ, Tiwari HK, Gore MA, Bradbury PJ, Yu JM, Arnett DK, Ordovas JM (2010). Mixed linear model approach adapted for genome-wide association studies. Nat Genet.

[45] Lü H-Y, Liu X-F, Wei S-P, Zhang Y-M (2011). Epistatic association mapping in homozygous crop cultivars. PLoS One.

[46] Zhang F-T, Zhu Z-H, Tong X-R, Zhu Z-X, Qi T, Zhu J (2015). Mixed linear model approaches of association mapping for complex traits based on omics variants. Sci Rep.

[47] Lou XY, Chen GB, Yan L, Ma JZ, Zhu J, Elston RC, Li MD (2007). A generalized combinatorial approach for detecting gene-by-gene and gene-by-environment interactions with application to nicotine dependence. Am J Hum Genet.

[48] Zhu Z, Tong X, Zhu Z, Liang M, Cui W, Su K, Li MD, Zhu J (2013). Development of GMDR-GPU for gene–gene interaction analysis and its application to WTCCC GWAS data for type 2 diabetes. PLoS One.

[49] Xu HM, Jiang BB, Cao YJ, Zhang YX, Zhan XD, Shen XH, Cheng SH, Lou XY, Cao LY (2015). Detection of epistatic and gene-environment interactions underlying three quality traits in Rice using high-throughput genome-wide data. Biomed Res Int.

[50] Li C, Miao H, Wei L, Zhang T, Han X, Zhang H (2014). Association mapping of seed oil and protein content in Sesamum indicum L. using SSR markers.

[51] Liu Q, Singh S, Green A (2002). High-oleic and high-stearic cottonseed oils: nutritionally improved cooking oils developed using gene silencing. J Am Coll Nutr.

[52] Chapman KD, Neogi PB, Hake KD, Stawska AA, Speed TR, Cotter MQ, Garrett DC, Kerby T, Richardson CD, Ayre BG (2008). Reduced oil accumulation in cottonseeds transformed with a nonfunctional allele of a Delta-12 fatty acid desaturase (). Crop Sci.

[53] Wang YH, Zhang HY, Zhang CL, Chen H, Fang XT, Zhang YS, Hou SS (2012). The fear gene Stathmin alleles generated Heterosis on feed efficiency parameters in Peking ducks. Anim Biotechnol.

[54] Raj A, Stephens M, Pritchard JK (2014). fastSTRUCTURE: variational inference of population structure in large SNP data sets. Genetics.

