# Supplementary figures and images for: Dissection of complicate genetic architecture and breeding perspective of cottonseed traits by genome-wide association study
Source: BMC Genomics. 2018 Jun 13;19:451. doi: 10.1186/s12864-018-4837-0 (PMC5998501; doi:10.1186/s12864-018-4837-0)

**
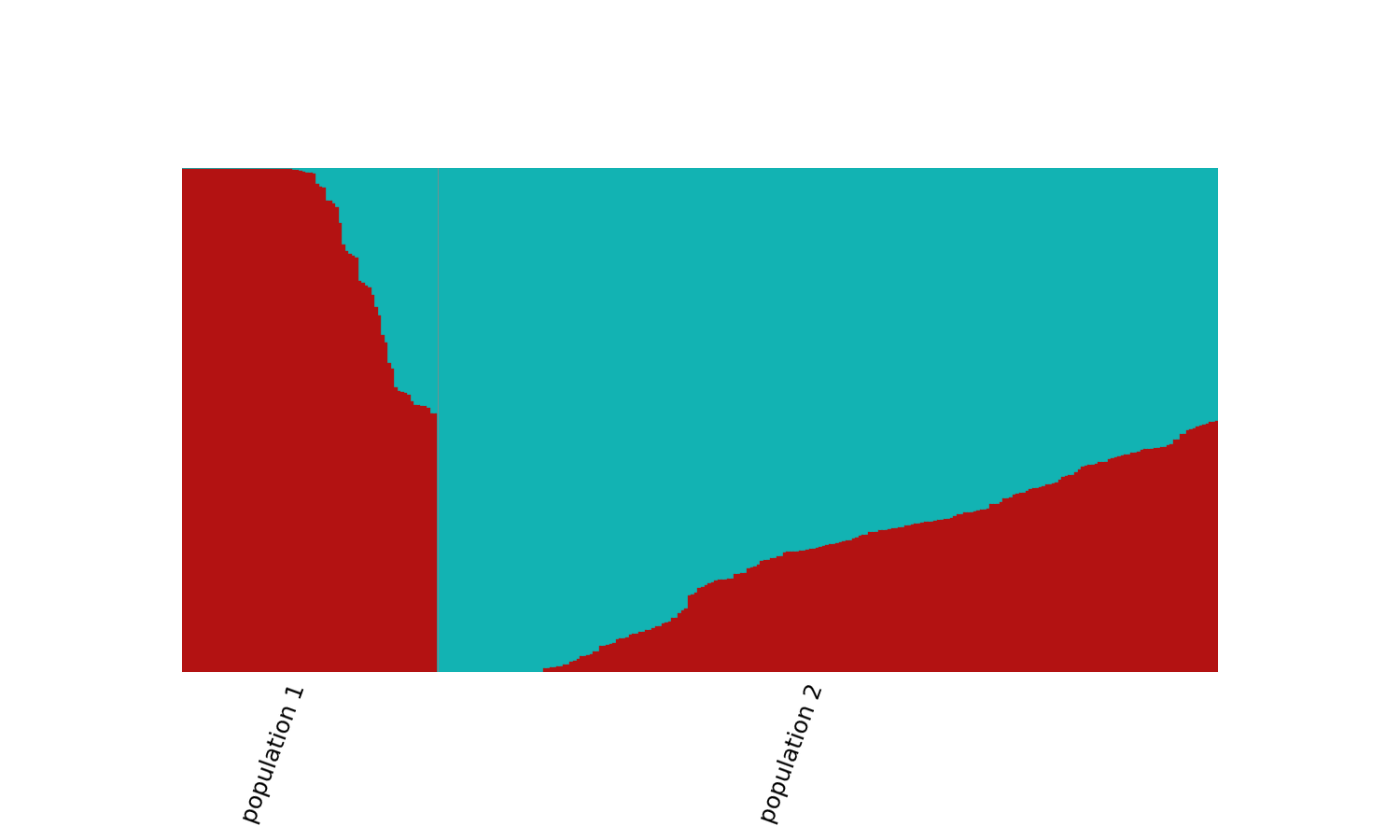
**

**Fig. S2.** Population structure of 316 cotton accessions based on pruned unlinked SNPs.

Supplement: Supplementary file 1 — Figure S2. Population structure of 316 cotton accessions based on pruned unlinked SNPs. (DOC 69 kb) [file 12864_2018_4837_MOESM1_ESM.doc]

**
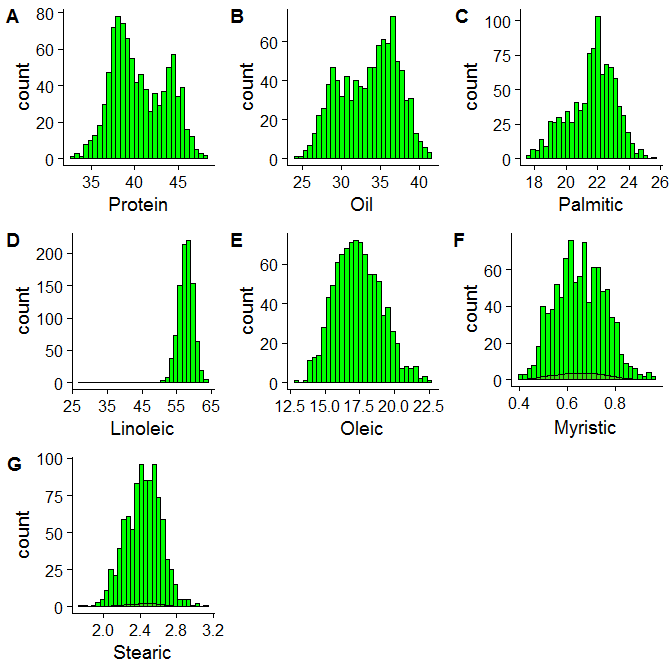
**

**Fig. S1.** The observed phenotype distribution of seven traits.

Supplement: Supplementary file 8 — Figure S1. The observed phenotype distribution of seven traits. (DOC 83 kb) [file 12864_2018_4837_MOESM8_ESM.doc]
