# Supplementary material for: Dissection of complicate genetic architecture and breeding perspective of cottonseed traits by genome-wide association study
Source: BMC Genomics. 2018 Jun 13;19:451. doi: 10.1186/s12864-018-4837-0 (PMC5998501; doi:10.1186/s12864-018-4837-0)
Supplement: Supplementary file 2 — Figure S3. Genome-wide average LD decay estimated based on the population of 316 cotton accessions. (DOC 58 kb) [file 12864_2018_4837_MOESM2_ESM.doc]

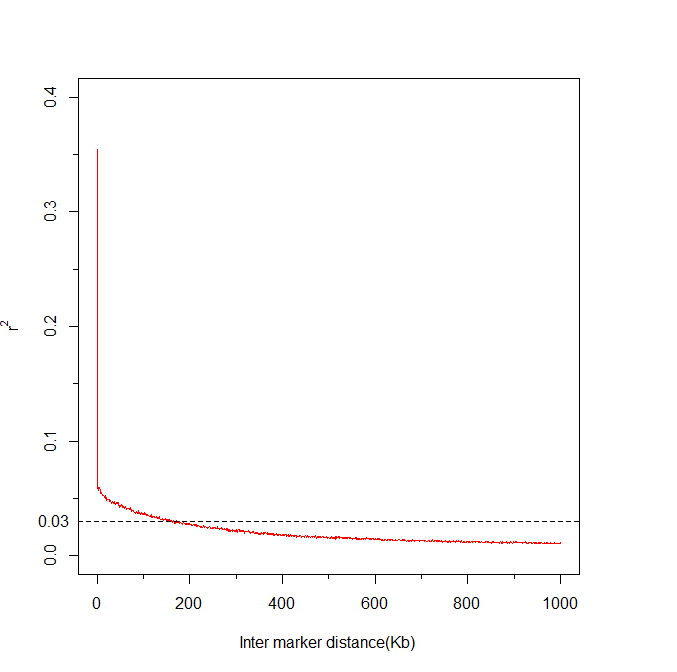


**Fig. S3.** Genome-wide average LD decay estimated based on the population of 316 cotton accessions. The LD decay rate measured by the average pairwise correlation coefficient (*r*^2^) drops to the half of its maximum value at the distance of ~160kb.
