# Supplementary material for: Dissection of complicate genetic architecture and breeding perspective of cottonseed traits by genome-wide association study
Source: BMC Genomics. 2018 Jun 13;19:451. doi: 10.1186/s12864-018-4837-0 (PMC5998501; doi:10.1186/s12864-018-4837-0)
Supplement: Supplementary file 3 — Table S3. The other genome-wide significant QTSs associated with five fatty acids. (DOC 185 kb) [file 12864_2018_4837_MOESM3_ESM.doc]

**Table S3.** The other genome-wide significant QTSs associated with five fatty acids

| Trait | QTS | ^a^Chr. | ^b^Allele^a^ | ^c^Effect type | Predict value | $-\log_{10}p$ | *h^2^*(*%*) |
| --- | --- | --- | --- | --- | --- | --- | --- |
| Protein | A5_65699627 | A5 | A/C | *a* | -0.265 | 4.06 | 0.21 |
|  | A6_4859753 | A6 | T/A | *a* | 0.217 | 2.22 | 0.14 |
|  | D5_26721498 | D5 | C/T | *a* | -0.274 | 4.23 | 0.23 |
|  |  | D5 | C/T | *d* | 1.827 | 3.72 | 5.06 |
|  | D8_2784522 | D8 | C/A | *a* | 0.337 | 5.84 | 0.34 |
|  |  | D8 | C/A | *d* | -0.524 | 1.34 | 0.42 |
|  | A5_22579901 & A5_65699627 | A5 & A5 | A/C & A/C | *da* | 0.825 | 2.79 | 2.06 |
|  | A5_65699627 & D6_58640083 | A5 & D6 | A/C & G/A | *aa* | -0.343 | 5.98 | 0.71 |
|  |  | A5 & D6 | A/C & G/A | *ad* | -0.58 | 1.75 | 1.02 |
|  | A11_27630663 & A11_115510024 | A11 & A11 | A/G & A/G | *aa* | 0.244 | 3.52 | 0.36 |
| Oil | A3_100487624 | A3 | G/A | *a* | -0.302 | 3.48 | 0.25 |
|  |  | A3 | G/A | *d* | -0.624 | 5.25 | 0.53 |
|  | A4_96357317 | A4 | A/G | *a* | 0.334 | 5.51 | 0.30 |
|  | A6_10405461 | A6 | T/C | *a* | 0.373 | 5.52 | 0.38 |
|  |  | A6 | T/C | *d* | -0.843 | 6.72 | 0.97 |
|  | A7_83990870 | A7 | A/G | *a* | -0.324 | 5.07 | 0.29 |
|  | A8_1041 | A8 | A/G | *a* | -0.291 | 4.3 | 0.23 |
|  | A8_47812698 | A8 | C/G | *a* | 0.321 | 4.75 | 0.28 |
|  |  | A8 | C/G | *d* | 0.962 | 3.93 | 1.26 |
|  | A11_34775904 | A11 | G/A | *a* | -0.185 | 1.89 | 0.09 |
|  |  | A11 | G/A | *d* | 1.197 | 5.18 | 1.95 |
|  | D1_62433793 | D1 | A/G | *a* | -0.371 | 6.25 | 0.37 |
|  |  | D1 | A/G | *d* | 1.123 | 4.27 | 1.71 |
|  | D2_38430405 | D2 | G/A | *a* | -0.291 | 4.32 | 0.23 |
|  | D6_35032171 | D6 | C/T | *a* | -0.324 | 5.21 | 0.29 |
|  | D9_37961611 | D9 | C/T | *a* | 0.154 | 1.42 | 0.06 |
|  |  | D9 | C/T | *d* | 0.778 | 2.56 | 0.82 |
|  | A8_1041 & D2_38430405 | A8 & D2 | A/G & G/A | *aa* | 0.264 | 3.64 | 0.38 |
| Oleic | A6_17433433 | A6 | G/A | *a* | -0.137 | 4.22 | 0.73 |
|  | A9_24122170 | A9 | C/A | *a* | 0.143 | 5.09 | 0.79 |
|  |  | A9 | C/A | *d* | 0.395 | 1.6 | 3.05 |
|  | A13_109599981 | A13 | A/C | *a* | -0.148 | 5.4 | 0.86 |
|  | D3_29047260 | D3 | T/C | *a* | 0.119 | 3.48 | 0.56 |
|  |  | D3 | T/C | *ae3* | -0.106 | 1.37 | 0.43 |
|  | D4_21291786 | D4 | G/A | *a* | 0.091 | 2.34 | 0.32 |
|  |  | D4 | G/A | *d* | -0.649 | 3.93 | 8.22 |
|  | D6_55114427 | D6 | T/C | *a* | 0.162 | 6.31 | 1.03 |
|  |  | D6 | T/C | *ae1* | 0.112 | 1.57 | 0.49 |
|  | D10_33120729 | D10 | G/A | *a* | -0.105 | 2.49 | 0.43 |
|  | D12_6151404 | D12 | T/C | *a* | 0.098 | 2.68 | 0.38 |
|  |  | D12 | T/C | *d* | 0.382 | 1.51 | 2.84 |
|  | D12_40866207 | D12 | C/T | *a* | 0.115 | 3.51 | 0.51 |
| Linoleic | A4_16656838 | A4 | G/A | *a* | -0.161 | 3.61 | 0.40 |
|  |  | A4 | G/A | *d* | -0.763 | 2.77 | 4.45 |
|  | A5_99301152 | A5 | G/T | *a* | 0.226 | 6.25 | 0.78 |
|  |  | A5 | G/T | *d* | -0.512 | 3.06 | 2.00 |
|  | A6_26471461 | A6 | A/G | *a* | -0.211 | 4.57 | 0.68 |
|  |  | A6 | A/G | *ae1* | 0.179 | 1.52 | 0.49 |
|  |  | A6 | A/G | *ae2* | -0.275 | 3.06 | 1.16 |
|  | A8_37826218 | A8 | C/A | *a* | -0.124 | 2.28 | 0.24 |
|  |  | A8 | C/A | *d* | -0.564 | 2.43 | 2.43 |
|  | A12_55865035 | A12 | T/C | *d* | -0.681 | 6.43 | 3.54 |
|  | A13_139041182 | A13 | T/C | *a* | -0.161 | 2.79 | 0.40 |
|  | D2_22616909 | D2 | A/C | *a* | 0.142 | 2.64 | 0.31 |
|  |  | D2 | A/C | *d* | -0.309 | 2.06 | 0.73 |
|  | D2_41368478 | D2 | T/G | *a* | -0.177 | 4 | 0.48 |
|  | D3_1889546 | D3 | T/C | *d* | -1.187 | 5.97 | 10.76 |
|  |  | D3 | T/C | *ae1* | -0.173 | 1.85 | 0.46 |
|  | D3_4982439 | D3 | G/A | *a* | -0.165 | 3.84 | 0.41 |
|  | D3_29047260 | D3 | T/C | *d* | -0.537 | 4.22 | 2.20 |
|  |  | D3 | T/C | *de3* | 0.403 | 1.31 | 1.24 |
|  | D7_54311632 | D7 | T/C | *a* | -0.161 | 3.58 | 0.40 |
|  |  | D7 | T/C | *d* | 0.442 | 1.33 | 1.49 |
|  | D8_17578248 | D8 | G/A | *a* | 0.104 | 1.69 | 0.17 |
|  |  | D8 | G/A | *d* | 0.789 | 6.07 | 4.76 |
|  | D9_7944 | D9 | C/T | *a* | 0.225 | 6.67 | 0.77 |
| Myristic | A3_34963031 | A3 | A/G | *a* | 0.005 | 1.82 | 0.32 |
|  | A3_122451703 | A3 | G/T | *a* | 0.009 | 4 | 0.93 |
|  |  | A3 | G/T | *d* | 0.022 | 3.38 | 3.06 |
|  | A4_16656838 | A4 | G/A | *a* | 0.009 | 4.89 | 1.07 |
|  | A4_68560523 | A4 | T/C | *a* | 0.011 | 6.97 | 1.54 |
|  | A5_42092939 | A5 | C/T | *a* | -0.009 | 4.31 | 0.90 |
|  | A6_26471461 | A6 | A/G | *a* | 0.006 | 1.71 | 0.40 |
|  |  | A6 | A/G | *ae2* | 0.009 | 1.81 | 1.07 |
|  | A6_117102987 | A6 | T/C | *a* | 0.005 | 1.36 | 0.25 |
|  |  | A6 | T/C | *d* | 0.027 | 5.43 | 4.61 |
|  | A11_78425154 | A11 | A/G | *a* | 0.011 | 6.27 | 1.38 |
|  | A12_96199502 | A12 | T/C | *a* | -0.011 | 6.38 | 1.40 |
|  | A12_117532407 | A12 | A/G | *a* | 0.011 | 6.7 | 1.47 |
|  | A13_128192242 | A13 | G/A | *a* | -0.007 | 3.01 | 0.63 |
|  | D1_53049670 | D1 | C/T | *a* | 0.008 | 3.01 | 0.83 |
|  |  | D1 | C/T | *d* | 0.013 | 2.97 | 1.02 |
|  | D2_48607576 | D2 | A/C | *a* | 0.01 | 5.42 | 1.25 |
|  | D3_4525316 | D3 | A/C | *a* | 0.007 | 2.67 | 0.51 |
|  | D8_40709742 | D8 | C/A | *a* | 0.005 | 1.76 | 0.33 |
|  |  | D8 | C/A | *d* | 0.035 | 5.12 | 7.51 |
|  | D9_7944 | D9 | C/T | *a* | -0.01 | 5.29 | 1.13 |
|  | D12_24367937 | D12 | C/A | *a* | 0.011 | 5.54 | 1.41 |
|  |  | D12 | C/A | *d* | -0.013 | 1.84 | 1.07 |
|  | A3_58421047 & A12_117532407 | A3 & A12 | G/T & A/G | *aae2* | 0.01 | 2.31 | 2.24 |
| Stearic | A1_7626295 | A1 | C/A | *a* | -0.011 | 1.71 | 0.35 |
|  |  | A1 | C/A | *d* | -0.043 | 2.07 | 2.85 |
|  | A2_25310067 | A2 | C/T | *a* | 0.024 | 5.4 | 1.69 |
|  |  | A2 | C/T | *ae1* | 0.022 | 2.04 | 1.42 |
|  |  | A2 | C/T | *de2* | 0.03 | 1.45 | 1.37 |
|  |  | A2 | C/T | *de3* | -0.033 | 1.69 | 1.67 |
|  | A4_52108283 | A4 | C/T | *a* | -0.025 | 6.06 | 1.94 |
|  |  | A4 | C/T | *d* | -0.019 | 1.49 | 0.55 |
|  | A4_135747886 | A4 | C/G | *a* | -0.01 | 1.39 | 0.33 |
|  |  | A4 | C/G | *ae2* | -0.018 | 1.47 | 0.97 |
|  |  | A4 | C/G | *ae3* | 0.03 | 3.36 | 2.66 |
|  | A7_40037862 | A7 | T/C | *d* | -0.045 | 4.06 | 3.03 |
|  | A7_115780789 | A7 | C/T | *a* | 0.018 | 4.27 | 0.98 |
|  | A11_32647777 | A11 | G/A | *a* | 0.019 | 3.93 | 1.09 |
|  |  | A11 | G/A | *ae3* | 0.017 | 1.57 | 0.87 |
|  | A12_78651650 | A12 | C/T | *a* | -0.017 | 3.31 | 0.86 |
|  |  | A12 | C/T | *ae2* | 0.03 | 3.58 | 2.65 |
|  |  | A12 | C/T | *ae3* | -0.033 | 4.19 | 3.18 |
|  | A13_21415280 | A13 | G/A | *a* | 0.014 | 2.87 | 0.62 |
|  |  | A13 | G/A | *ae3* | 0.019 | 2.1 | 1.09 |
|  | A13_33729709 | A13 | T/C | *d* | 0.033 | 2.87 | 1.62 |
|  |  | A13 | T/C | *ae1* | -0.016 | 1.45 | 0.80 |
|  | A13_55888152 | A13 | C/T | *a* | -0.017 | 2.71 | 0.82 |
|  |  | A13 | C/T | *d* | 0.023 | 2.35 | 0.81 |
|  | A13_123882086 | A13 | G/T | *a* | 0.02 | 4.89 | 1.22 |
|  |  | A13 | G/T | *d* | 0.085 | 6 | 10.78 |
|  | D3_6711938 | D3 | C/T | *a* | 0.024 | 5.26 | 1.74 |
|  |  | D3 | C/T | *d* | 0.035 | 4.61 | 1.85 |
|  | D5_44746794 | D5 | A/G | *a* | 0.015 | 2.99 | 0.71 |
|  |  | D5 | A/G | *ae3* | -0.018 | 1.84 | 1.00 |
|  | D7_45595959 | D7 | A/G | *a* | 0.022 | 5.84 | 1.50 |
|  |  | D7 | A/G | *ae3* | -0.015 | 1.45 | 0.68 |
|  | D8_50516428 | D8 | T/C | *a* | -0.018 | 3.46 | 1.01 |
|  | D10_5643096 | D10 | T/A | *ae1* | 0.027 | 3.43 | 2.11 |
|  |  | D10 | T/A | *ae3* | -0.018 | 1.81 | 0.97 |
|  | D12_44593838 | D12 | A/G | *a* | 0.013 | 1.9 | 0.52 |
|  |  | D12 | A/G | *d* | -0.035 | 4.63 | 1.88 |

^a^ Chromosome. A represents A genome of cotton and D represents D genome of cotton. ^b^(Major allele/minor allele). ^c^ *a*, *d*, *aa*, *ad*, *da* and *dd* denote the additive effect, the dominant effect, the epistasis effects of the additive × additive, the additive × dominance, the dominance × additive, the dominance × dominance respectively; *ae1*, *ae2* and *ae*3 denote the interaction effects of the additive with the first, the second and the third environments respectively; *de2*, *de3* denote the interaction effects of the dominance with the second and the third environment; *aae2*, and *ade2* denote the interaction of the additive × additive by environment 2 and the additive × dominance by environment 2 respectively; *h*^2^ is the heritability in percentage due to each genetic effect of QTS.
