# Supplementary material for: Dissection of complicate genetic architecture and breeding perspective of cottonseed traits by genome-wide association study
Source: BMC Genomics. 2018 Jun 13;19:451. doi: 10.1186/s12864-018-4837-0 (PMC5998501; doi:10.1186/s12864-018-4837-0)
Supplement: Supplementary file 4 — Table S2. Phenotypic and genotypic correlation between seven cottonseed traits. (DOC 58 kb) [file 12864_2018_4837_MOESM4_ESM.doc]

**Table S2.** Phenotypic and genotypic correlation between seven cottonseed traits

|  | Protein | Oil | Palmitic | Oleic | Linoleic | Myristic | Stearic |
| --- | --- | --- | --- | --- | --- | --- | --- |
| Protein | 1 | -0.75^**^ | -0.11 | -0.28^**^ | 0.21^**^ | -0.14 | -0.01 |
| Oil | -0.63^**^ | 1 | 0.14 | 0.30^**^ | -0.35^**^ | 0.11 | 0.02 |
| Palmitic | -0.09^**^ | 0.09^**^ | 1 | -0.24^**^ | -0.61^**^ | 0.82^**^ | -0.14 |
| Oleic | -0.31^**^ | 0.12^**^ | -0.27^**^ | 1 | -0.53^**^ | -0.05 | 0.05 |
| Linoleic | 0.30^**^ | -0.11^**^ | -0.50^**^ | -0.54^**^ | 1 | -0.59^**^ | -0.03 |
| Myristic | -0.14^**^ | 0.05 | 0.65^**^ | 0.21^**^ | -0.59^**^ | 1 | -0.22^**^ |
| Stearic | -0.06 | -0.08^**^ | -0.02 | 0.17^**^ | -0.27^**^ | 0.01 | 1 |

The upper-triangle part is the genotypic correlation coefficients, and the lower-triangle part is the phenotypic correlation coefficients. ^**^, *P* < 0.01; ^*^, *P* < 0.05.
