# Supplementary material for: Dissection of complicate genetic architecture and breeding perspective of cottonseed traits by genome-wide association study
Source: BMC Genomics. 2018 Jun 13;19:451. doi: 10.1186/s12864-018-4837-0 (PMC5998501; doi:10.1186/s12864-018-4837-0)
Supplement: Supplementary file 5 — Table S4. Predicted genetic values (G) for QQ, qq, Qq, superior homozygous line (SL), and superior hybrids (SH) of seven cottonseed traits. (DOC 114 kb) [file 12864_2018_4837_MOESM5_ESM.doc]

**Table S4. Predicted genetic values (G) for QQ, qq, Qq, superior homozygous line (SL), and superior hybrids (SH) of seven cottonseed traits**

| Trait | Genotype | G | G1 | G2 | G3 |
| --- | --- | --- | --- | --- | --- |
| Protein | QQ | 1.682 | 1.682 | 1.682 | 1.682 |
|  | Qq | -2.962 | -2.962 | -2.962 | -2.962 |
|  | SL(+) | 6.693 | 6.693 | 6.693 | 6.693 |
|  | SL(-) | -6.048 | -6.048 | -6.048 | -6.048 |
|  | Qq | -0.091 | -0.091 | -0.091 | -0.091 |
|  | SH(+) | 8.246 | 8.246 | 8.246 | 8.246 |
|  | SH(-) | -8.712 | -8.712 | -8.712 | -8.712 |
| Oil | QQ | -2.415 | -2.415 | -2.415 | -2.415 |
|  | qq | -0.397 | -0.397 | -0.397 | -0.397 |
|  | SL(+) | 6.857 | 6.857 | 6.857 | 6.857 |
|  | SL(-) | -8.818 | -8.818 | -8.818 | -8.818 |
|  | Qq | 0.213 | 0.213 | -1.743 | 1.475 |
|  | SH(+) | 12.656 | 12.656 | 12.656 | 12.656 |
|  | SH(-) | -13.903 | -13.903 | -14.684 | -13.903 |
| Oleic | QQ | 0.420 | 0.532 | 0.420 | 0.315 |
|  | qq | -0.792 | -0.904 | -0.792 | -0.686 |
|  | SL(+) | 3.287 | 3.399 | 3.287 | 3.182 |
|  | SL(-) | -3.099 | -3.211 | -3.099 | -2.993 |
|  | Qq | 1.215 | 1.215 | 1.215 | 1.215 |
|  | SH(+) | 5.567 | 5.678 | 5.567 | 5.461 |
|  | SH(-) | -4.395 | -4.507 | -4.395 | -4.290 |
| Linoleic | QQ | -1.171 | -1.165 | -1.446 | -1.171 |
|  | qq | 0.850 | 0.845 | 1.125 | 0.850 |
|  | SL(+) | 3.782 | 3.776 | 4.057 | 3.782 |
|  | SL(-) | -3.782 | -3.776 | -4.057 | -3.782 |
|  | Qq | -1.226 | -1.226 | -1.226 | -0.823 |
|  | SH(+) | 5.430 | 5.425 | 5.705 | 5.430 |
|  | SH(-) | -8.138 | -7.959 | -8.413 | -7.736 |
| Palmitic | QQ | -0.557 | -0.557 | -0.557 | -0.557 |
|  | qq | 0.118 | 0.118 | 0.118 | 0.118 |
|  | SL(+) | 2.190 | 2.190 | 2.190 | 2.190 |
|  | SL(-) | -2.629 | -2.629 | -2.629 | -2.629 |
|  | Qq | 2.412 | 2.412 | 2.412 | 2.412 |
|  | SH(+) | 3.263 | 3.263 | 3.263 | 3.263 |
|  | SH(-) | -3.100 | -3.100 | -3.100 | -3.100 |
| Myristic | QQ | 0.050 | 0.050 | 0.068 | 0.050 |
|  | qq | -0.050 | -0.050 | -0.049 | -0.050 |
|  | SL(+) | 0.241 | 0.241 | 0.260 | 0.241 |
|  | SL(-) | -0.241 | -0.241 | -0.260 | -0.241 |
|  | Qq | 0.014 | 0.014 | 0.014 | 0.014 |
|  | SH(+) | 0.332 | 0.332 | 0.351 | 0.332 |
|  | SH(-) | -0.279 | -0.279 | -0.297 | -0.279 |
| Stearic | QQ | 0.089 | 0.057 | 0.030 | 0.196 |
|  | qq | -0.089 | -0.057 | -0.030 | -0.196 |
|  | SL(+) | 0.334 | 0.428 | 0.385 | 0.527 |
|  | SL(-) | -0.334 | -0.428 | -0.385 | -0.527 |
|  | Qq | 0.033 | 0.033 | 0.063 | 0.035 |
|  | SH(+) | 0.449 | 0.527 | 0.506 | 0.641 |
|  | SH(-) | -0.433 | -0.528 | -0.485 | -0.636 |

QQ and qq denote the homozygous genotypes composed of the major alleles and the minor alleles at each QTS, respectively, Qq denotes the heterozygous genotypes composed of the major and the minor allele at each QTS; G denotes the genotypic value estimated by genetic main effects of QTSs, G*i*,(*i*=1, 2, 3) is the genotypic values estimated by the main effects and the environment-specific interaction effects of each QTS; SL(+)/(-) denotes the superior homozygous line achieving the maximum/the minimum in all designed homozygous lines; SH(+)/(-) denotes the superior hybrid achieving the maximum/the minimum in all designed hybrids.
