# Supplementary material for: Dissection of complicate genetic architecture and breeding perspective of cottonseed traits by genome-wide association study
Source: BMC Genomics. 2018 Jun 13;19:451. doi: 10.1186/s12864-018-4837-0 (PMC5998501; doi:10.1186/s12864-018-4837-0)
Supplement: Supplementary file 6 — Table S5. The SNP genotypes achieving the maximum genetic value of seed traits in designed superior homozygous lines and hybrids at the QTSs absent in the Table 5. (DOC 168 kb) [file 12864_2018_4837_MOESM6_ESM.doc]

**Table S5. The genotypes achieving the maximum genetic value in designed superior homozygous lines and hybrids at the QTSs absent in the Table 5**

| Trait | QTS | GSL(+) | SL(+)1 | SL(+)2 | SL(+)3 | GSH(+) | SH(+)1 | SH(+)2 | SH(+)3 |
| --- | --- | --- | --- | --- | --- | --- | --- | --- | --- |
| Protein | A3_115443958 | TT | TT | TT | TT | TT | TT | TT | TT |
|  | A5_65699627 | CC | CC | CC | CC | CC | CC | CC | CC |
|  | A6_4859753 | TT | TT | TT | TT | TT | TT | TT | TT |
|  | A6_29542325 | GG | GG | GG | GG | GG | GG | GG | GG |
|  | A11_27630663 | AA | AA | AA | AA | AA | AA | AA | AA |
|  | A11_115510024 | AA | AA | AA | AA | AA | AA | AA | AA |
|  | D2_383581 | CC | CC | CC | CC | CC | CC | CC | CC |
|  | D5_26721498 | TT | TT | TT | TT | CT | CT | CT | CT |
|  | D6_58640083 | GG | GG | GG | GG | GG | GG | GG | GG |
|  | D8_2784522 | CC | CC | CC | CC | CC | CC | CC | CC |
|  | D8_18888997 | GG | GG | GG | GG | GG | GG | GG | GG |
| Oil | A4_96357317 | AA | AA | AA | AA | AA | AA | AA | AA |
|  | A6_10405461 | TT | TT | TT | TT | TT | TT | TT | TT |
|  | A6_124107263 | GG | GG | GG | GG | GG | GG | GG | GG |
|  | A7_83990870 | GG | GG | GG | GG | GG | GG | GG | GG |
|  | A7_110987422 | AA | AA | AA | AA | AC | AC | AC | AC |
|  | A8_1041 | GG | GG | GG | GG | GG | GG | GG | GG |
|  | A8_47812698 | CC | CC | CC | CC | CG | CG | CG | CG |
|  | A9_85081637 | CC | CC | CC | CC | TC | TC | TC | TC |
|  | A11_34775904 | AA | AA | AA | AA | GA | GA | GA | GA |
|  | A13_83121382 | TT | TT | TT | TT | TT | TT | TT | TT |
|  | D2_38430405 | AA | AA | AA | AA | AA | AA | AA | AA |
|  | D6_35032171 | TT | TT | TT | TT | TT | TT | TT | TT |
|  | D6_54108367 | GG | GG | GG | GG | GA | GA | GA | GA |
|  | D9_37961611 | CC | CC | CC | CC | CT | CT | CT | CT |
|  | D10_18219333 | CC | CC | CC | CC | CC | CC | CC | CC |
|  | D12_3388946 | GG | GG | GG | GG | GG | GG | GG | GG |
|  | D12_41865508 | GG | GG | GG | GG | GA | GA | GA | GA |
| Oleic | A1_44951529 | GG | GG | GG | GG | GG | GG | GG | GG |
|  | A1_85724143 | CC | CC | CC | CC | CC | CC | CC | CC |
|  | A3_4717931 | CC | CC | CC | CC | CC | CC | CC | CC |
|  | A6_17433433 | AA | AA | AA | AA | AA | AA | AA | AA |
|  | A9_24122170 | CC | CC | CC | CC | CA | CA | CA | CA |
|  | A13_109599981 | CC | CC | CC | CC | CC | CC | CC | CC |
|  | D1_37367501 | AA | AA | AA | AA | AA | AA | AA | AA |
|  | D5_24437741 | AA | AA | AA | AA | AA | AA | AA | AA |
|  | D5_31125264 | AA | AA | AA | AA | AA | AA | AA | AA |
|  | D6_55114427 | TT | TT | TT | TT | TT | TT | TT | TT |
|  | D9_148268 | CC | CC | CC | CC | CC | CC | CC | CC |
|  | D9_45944489 | TT | TT | TT | TT | TT | TT | TT | TT |
|  | D10_33120729 | AA | AA | AA | AA | AA | AA | AA | AA |
|  | D12_6151404 | TT | TT | TT | TT | TC | TC | TC | TC |
|  | D12_40866207 | CC | CC | CC | CC | CC | CC | CC | CC |
| Linoleic | A5_99301152 | GG | GG | GG | GG | GG | GG | GG | GG |
|  | A6_69314946 | GG | GG | GG | GG | GG | GG | GG | GG |
|  | A8_37826218 | AA | AA | AA | AA | AA | AA | AA | AA |
|  | A9_23536520 | GG | GG | GG | GG | GG | GG | GG | GG |
|  | A12_55865035 | TT | TT | TT | TT | TT | TT | TT | TT |
|  | A13_29883619 | TT | TT | TT | TT | TT | TT | TT | TT |
|  | A13_139041182 | CC | CC | CC | CC | CC | CC | CC | CC |
|  | D2_22616909 | AA | AA | AA | AA | AA | AA | AA | AA |
|  | D2_41368478 | GG | GG | GG | GG | GG | GG | GG | GG |
|  | D3_4982439 | AA | AA | AA | AA | AA | AA | AA | AA |
|  | D6_59379832 | CC | CC | CC | CC | AC | AC | AC | AC |
|  | D7_54311632 | CC | CC | CC | CC | TC | TC | TC | TC |
|  | D8_17578248 | GG | GG | GG | GG | GA | GA | GA | GA |
| Palmitic | A1_61493378 | TT | TT | TT | TT | TG | TG | TG | TG |
|  | A11_600080 | AA | AA | AA | AA | AA | AA | AA | AA |
|  | A13_119809048 | GG | GG | GG | GG | GA | GA | GA | GA |
|  | D6_10836500 | CC | CC | CC | CC | CC | CC | CC | CC |
| Myristic | A1_35871478 | TT | TT | TT | TT | TT | TT | TT | TT |
|  | A1_54364850 | TT | TT | TT | TT | TT | TT | TT | TT |
|  | A1_67270927 | CC | CC | CC | CC | CC | CC | CC | CC |
|  | A3_34963031 | AA | AA | AA | AA | AA | AA | AA | AA |
|  | A3_58421047 | GG | GG | GG | GG | GG | GG | GG | GG |
|  | A3_122451703 | GG | GG | GG | GG | GT | GT | GT | GT |
|  | A4_68560523 | TT | TT | TT | TT | TT | TT | TT | TT |
|  | A5_42092939 | TT | TT | TT | TT | TT | TT | TT | TT |
|  | A6_117102987 | TT | TT | TT | TT | TC | TC | TC | TC |
|  | A8_86207865 | AA | AA | AA | AA | AA | AA | AA | AA |
|  | A11_78425154 | AA | AA | AA | AA | AA | AA | AA | AA |
|  | A12_37853320 | GG | GG | GG | GG | GA | GA | GA | GA |
|  | A12_96199502 | CC | CC | CC | CC | CC | CC | CC | CC |
|  | A12_117532407 | AA | AA | AA | AA | AA | AA | AA | AA |
|  | A13_19834182 | CC | CC | CC | CC | CC | CC | CC | CC |
|  | D1_53049670 | CC | CC | CC | CC | CT | CT | CT | CT |
|  | D2_48607576 | AA | AA | AA | AA | AA | AA | AA | AA |
|  | D3_4525316 | AA | AA | AA | AA | AA | AA | AA | AA |
|  | D8_40709742 | CC | CC | CC | CC | CA | CA | CA | CA |
|  | D12_24367937 | CC | CC | CC | CC | CC | CC | CC | CC |
| Stearic | A1_7626295 | AA | AA | AA | AA | AA | AA | AA | AA |
|  | A4_52108283 | TT | TT | TT | TT | TT | TT | TT | TT |
|  | A4_135747886 | GG | GG | GG | CC | GG | GG | GG | CC |
|  | A7_40037862 | TT | TT | TT | TT | TT | TT | TT | TT |
|  | A7_115780789 | CC | CC | CC | CC | CC | CC | CC | CC |
|  | A11_110829220 | AA | AA | AA | AA | AA | AA | AA | AA |
|  | A12_78651650 | TT | TT | CC | TT | TT | TT | CC | TT |
|  | A13_21415280 | GG | GG | GG | GG | GG | GG | GG | GG |
|  | A13_55888152 | TT | TT | TT | TT | CT | CT | CT | CT |
|  | A13_123882086 | GG | GG | GG | GG | GT | GT | GT | GT |
|  | D3_6711938 | CC | CC | CC | CC | CT | CT | CT | CT |
|  | D5_44746794 | AA | AA | AA | GG | AA | AA | AA | GG |
|  | D7_45595959 | AA | AA | AA | AA | AA | AA | AA | AA |
|  | D8_50516428 | CC | CC | CC | CC | CC | CC | CC | CC |
|  | D10_5643096 | TT | TT | TT | AA | TT | TT | TT | AA |
|  | D10_30598593 | TT | GG | GG | TT | TT | GG | GG | TT |
|  | D12_44593838 | AA | AA | AA | AA | AA | AA | AA | AA |

GSL and GSH stand for the general superior homozygous line and the general superior hybrid without consideration of gene by environment interaction respectively; SL and SH stand for the environment-specific superior homozygous line and the environment-specific superior hybrid with consideration of gene by environment interaction respectively; the sign “+” in the parentheses indicates the genotype could achieve the maximum genetic value in all designed lines; the number 1,2, and 3 on the right of the parentheses are environment codes.
