# Supplementary material for: Dissection of complicate genetic architecture and breeding perspective of cottonseed traits by genome-wide association study
Source: BMC Genomics. 2018 Jun 13;19:451. doi: 10.1186/s12864-018-4837-0 (PMC5998501; doi:10.1186/s12864-018-4837-0)
Supplement: Supplementary file 7 — Table S6. The SNP genotypes achieving the minimum genetic value of seed traits in designed lines for QTSs (DOC 210 kb) [file 12864_2018_4837_MOESM7_ESM.doc]

**Table S6.** The genotypes achieving the minimum genetic value in designed lines for QTSs

| Trait | QTS | GSL(-) | SL(-)1 | SL(-)2 | SL(-)3 | GSH(-) | SH(-)1 | SH(-)2 | SH(-)3 |
| --- | --- | --- | --- | --- | --- | --- | --- | --- | --- |
| Protein | A3_115443958 | AA | AA | AA | AA | AA | AA | AA | AA |
|  | A5_65699627 | AA | AA | AA | AA | AA | AA | AA | AA |
|  | A6_4859753 | TT | TT | TT | TT | TT | TT | TT | TT |
|  | A6_29542325 | AA | AA | AA | AA | GA | GA | GA | GA |
|  | A7_1504479 | GG | GG | GG | GG | AG | AG | AG | AG |
|  | A11_27630663 | GG | GG | GG | GG | GG | GG | GG | GG |
|  | A11_115510024 | GG | GG | GG | GG | GG | GG | GG | GG |
|  | D1_616439 | CC | CC | CC | CC | CC | CC | CC | CC |
|  | D2_383581 | CC | CC | CC | CC | AC | AC | AC | AC |
|  | D3_35705563 | TT | TT | TT | TT | TT | TT | TT | TT |
|  | D5_26721498 | CC | CC | CC | CC | CC | CC | CC | CC |
|  | D6_58640083 | GG | GG | GG | GG | GA | GA | GA | GA |
|  | D8_2784522 | AA | AA | AA | AA | CA | CA | CA | CA |
|  | D8_18888997 | AA | AA | AA | AA | AA | AA | AA | AA |
|  | A5_22579901 | AA | AA | AA | AA | AA | AA | AA | AA |
| Oil | A2_58832915 | GG | GG | GG | GG | GA | GA | GA | GA |
|  | A3_100487624 | GG | GG | GG | GG | GG | GG | GG | GG |
|  | A4_96357317 | GG | GG | GG | GG | GG | GG | GG | GG |
|  | A6_10405461 | CC | CC | CC | CC | TC | TC | TC | TC |
|  | A6_124107263 | AA | AA | AA | AA | GA | GA | GA | GA |
|  | A7_83990870 | AA | AA | AA | AA | AA | AA | AA | AA |
|  | A7_110987422 | AA | AA | AA | AA | AC | AC | AC | AC |
|  | A8_1041 | AA | AA | AA | AA | AA | AA | AA | AA |
|  | A8_47812698 | GG | GG | GG | GG | GG | GG | GG | GG |
|  | A9_85081637 | TT | TT | TT | TT | TT | TT | TT | TT |
|  | A11_34775904 | GG | GG | GG | GG | GG | GG | GG | GG |
|  | A13_83121382 | CC | CC | CC | CC | CC | CC | TC | CC |
|  | D1_62433793 | AA | AA | AA | AA | AA | AA | AA | AA |
|  | D2_38430405 | GG | GG | GG | GG | GG | GG | GG | GG |
|  | D3_35705563 | TT | TT | TT | TT | TC | TC | TC | TC |
|  | D6_35032171 | CC | CC | CC | CC | CC | CC | CC | CC |
|  | D6_54108367 | GG | GG | GG | GG | GG | GG | GG | GG |
|  | D9_37961611 | CC | CC | CC | CC | CC | CC | CC | CC |
|  | D10_18219333 | CC | CC | CC | CC | CC | CC | CC | CC |
|  | D12_3388946 | AA | AA | AA | AA | AG | AG | AG | AG |
|  | D12_41865508 | AA | AA | AA | AA | AA | AA | AA | AA |
| Oleic | A1_44951529 | AA | AA | AA | AA | AA | AA | AA | AA |
|  | A1_85724143 | CC | CC | CC | CC | CC | CC | CC | CC |
|  | A3_4717931 | TT | TT | TT | TT | CT | CT | CT | CT |
|  | A6_17433433 | GG | GG | GG | GG | GG | GG | GG | GG |
|  | A7_2266630 | GG | GG | GG | GG | GG | GG | GG | GG |
|  | A9_24122170 | AA | AA | AA | AA | AA | AA | AA | AA |
|  | A12_120581335 | GG | GG | GG | GG | GG | GG | GG | GG |
|  | A13_109599981 | AA | AA | AA | AA | AA | AA | AA | AA |
|  | D1_1087912 | CC | CC | CC | CC | CC | CC | CC | CC |
|  | D1_37367501 | GG | GG | GG | GG | GG | GG | GG | GG |
|  | D3_1889546 | TT | TT | TT | TT | TT | TT | TT | TT |
|  | D3_29047260 | CC | CC | CC | CC | CC | CC | CC | CC |
|  | D4_21291786 | AA | AA | AA | AA | GA | GA | GA | GA |
|  | D5_24437741 | GG | GG | GG | GG | AG | AG | AG | AG |
|  | D5_31125264 | GG | GG | GG | GG | GA | GA | GA | GA |
|  | D6_55114427 | CC | CC | CC | CC | CC | CC | CC | CC |
|  | D9_148268 | TT | TT | TT | TT | TT | TT | TT | TT |
|  | D9_45944489 | CC | CC | CC | CC | CC | CC | CC | CC |
|  | D10_33120729 | GG | GG | GG | GG | GG | GG | GG | GG |
|  | D12_6151404 | CC | CC | CC | CC | CC | CC | CC | CC |
|  | D12_40866207 | TT | TT | TT | TT | TT | TT | TT | TT |
| Linoleic | A4_16656838 | GG | GG | GG | GG | GA | GA | GA | GA |
|  | A5_99301152 | TT | TT | TT | TT | GT | GT | GT | GT |
|  | A6_26471461 | AA | AA | AA | AA | AA | AA | AA | AA |
|  | A6_69314946 | TT | TT | TT | TT | TT | TT | TT | TT |
|  | A8_37826218 | CC | CC | CC | CC | CA | CA | CA | CA |
|  | A9_23536520 | AA | AA | AA | AA | AA | AA | AA | AA |
|  | A12_55865035 | TT | TT | TT | TT | TC | TC | TC | TC |
|  | A12_117532394 | GG | GG | GG | GG | GG | GG | GG | GG |
|  | A13_29883619 | CC | CC | CC | CC | TC | TC | TC | TC |
|  | A13_139041182 | TT | TT | TT | TT | TT | TT | TT | TT |
|  | D2_22616909 | CC | CC | CC | CC | AC | AC | AC | AC |
|  | D2_41368478 | TT | TT | TT | TT | TT | TT | TT | TT |
|  | D3_1889546 | TT | TT | TT | TT | TC | TC | TC | TC |
|  | D3_4982439 | GG | GG | GG | GG | GG | GG | GG | GG |
|  | D3_29047260 | TT | TT | TT | TT | TC | TC | TC | TC |
|  | D4_21291786 | GG | GG | GG | GG | GG | GG | GG | GG |
|  | D5_47644432 | GG | GG | GG | GG | GG | GG | GG | GG |
|  | D6_59379832 | AA | AA | AA | AA | AA | AA | AA | AA |
|  | D7_54311632 | TT | TT | TT | TT | TT | TT | TT | TT |
|  | D8_17578248 | GG | GG | GG | GG | GG | GG | GG | GG |
|  | D9_7944 | TT | TT | TT | TT | TT | TT | TT | TT |
| Palmitic | A1_61493378 | GG | GG | GG | GG | GG | GG | GG | GG |
|  | A7_642514 | CC | CC | CC | CC | CC | CC | CC | CC |
|  | A11_600080 | GG | GG | GG | GG | GG | GG | GG | GG |
|  | A12_117532394 | AA | AA | AA | AA | AA | AA | AA | AA |
|  | A13_119809048 | AA | AA | AA | AA | AA | AA | AA | AA |
|  | D4_10313468 | AA | AA | AA | AA | AC | AC | AC | AC |
|  | D5_66975738 | AA | AA | AA | AA | AA | AA | AA | AA |
|  | D6_10836500 | TT | TT | TT | TT | TT | TT | TT | TT |
| Myristic | A1_35871478 | CC | CC | CC | CC | CT | CT | CT | CT |
|  | A1_54364850 | CC | CC | CC | CC | CC | CC | CC | CC |
|  | A1_67270927 | TT | TT | TT | TT | TT | TT | TT | TT |
|  | A3_34963031 | GG | GG | GG | GG | GG | GG | GG | GG |
|  | A3_58421047 | GG | GG | GG | GG | GG | GG | GG | GG |
|  | A3_122451703 | TT | TT | TT | TT | TT | TT | TT | TT |
|  | A4_16656838 | AA | AA | AA | AA | AA | AA | AA | AA |
|  | A4_68560523 | CC | CC | CC | CC | CC | CC | CC | CC |
|  | A5_42092939 | CC | CC | CC | CC | CC | CC | CC | CC |
|  | A6_26471461 | GG | GG | GG | GG | GG | GG | GG | GG |
|  | A6_117102987 | CC | CC | CC | CC | CC | CC | CC | CC |
|  | A7_642514 | CC | CC | CC | CC | CT | CT | CT | CT |
|  | A8_86207865 | GG | GG | GG | GG | GG | GG | GG | GG |
|  | A11_78425154 | GG | GG | GG | GG | GG | GG | GG | GG |
|  | A12_37853320 | AA | AA | AA | AA | AA | AA | AA | AA |
|  | A12_96199502 | TT | TT | TT | TT | TT | TT | TT | TT |
|  | A12_117532407 | GG | GG | GG | GG | GG | GG | GG | GG |
|  | A13_19834182 | TT | TT | TT | TT | CT | CT | CT | CT |
|  | A13_128192242 | GG | GG | GG | GG | GG | GG | GG | GG |
|  | D1_53049670 | TT | TT | TT | TT | TT | TT | TT | TT |
|  | D2_48607576 | CC | CC | CC | CC | CC | CC | CC | CC |
|  | D3_4525316 | CC | CC | CC | CC | CC | CC | CC | CC |
|  | D8_40709742 | AA | AA | AA | AA | AA | AA | AA | AA |
|  | D9_7944 | CC | CC | CC | CC | CC | CC | CC | CC |
|  | D12_24367937 | AA | AA | AA | AA | CA | CA | CA | CA |
| Stearic | A1_7626295 | CC | CC | CC | CC | CA | CA | CA | CA |
|  | A2_25310067 | TT | TT | TT | TT | TT | TT | TT | CT |
|  | A4_17627308 | AA | GG | GG | AA | AA | GG | GG | AA |
|  | A4_52108283 | CC | CC | CC | CC | CC | CC | CC | CC |
|  | A4_135747886 | CC | CC | CC | GG | CC | CC | CC | GG |
|  | A7_40037862 | TT | TT | TT | TT | TC | TC | TC | TC |
|  | A7_115780789 | TT | TT | TT | TT | TT | TT | TT | TT |
|  | A11_32647777 | AA | AA | AA | AA | AA | AA | AA | AA |
|  | A11_110829220 | GG | GG | GG | GG | GG | GG | GG | GG |
|  | A12_78651650 | CC | CC | TT | CC | CC | CC | TT | CC |
|  | A13_21415280 | AA | AA | AA | AA | AA | AA | AA | AA |
|  | A13_33729709 | TT | TT | TT | TT | TT | TT | TT | TT |
|  | A13_55888152 | CC | CC | CC | CC | CC | CC | CC | CC |
|  | A13_123882086 | TT | TT | TT | TT | TT | TT | TT | TT |
|  | D1_10742184 | TT | TT | TT | TT | TT | TT | TT | TT |
|  | D3_6711938 | TT | TT | TT | TT | TT | TT | TT | TT |
|  | D5_44746794 | GG | GG | GG | AA | GG | GG | GG | AA |
|  | D7_45595959 | GG | GG | GG | GG | GG | GG | GG | GG |
|  | D8_50516428 | TT | TT | TT | TT | TT | TT | TT | TT |
|  | D10_5643096 | TT | AA | TT | TT | TT | AA | TT | TT |
|  | D10_30598593 | TT | TT | TT | GG | TT | TT | TT | GG |
|  | D12_44593838 | GG | GG | GG | GG | AG | AG | AG | AG |

GSL and GSH stand for the general superior homozygous line and the general superior hybrid without consideration of gene by environment interaction respectively; SL and SH stand for the environment-specific superior homozygous line and the environment-specific superior line with consideration of gene by environment interaction respectively; the sign “-” in the parentheses indicates the genotype could achieve the minimum genetic value in all designed lines; the number 1,2, and 3 on the right of parentheses are environment codes.
