# Supplementary material for: Dissection of complicate genetic architecture and breeding perspective of cottonseed traits by genome-wide association study
Source: BMC Genomics. 2018 Jun 13;19:451. doi: 10.1186/s12864-018-4837-0 (PMC5998501; doi:10.1186/s12864-018-4837-0)
Supplement: Supplementary file 9 — Table S1. The information of 316 accessions and the summary statistics of seven seed traits (DOC 321 kb) [file 12864_2018_4837_MOESM9_ESM.doc]

**Table S1. The information of 316 accessions and the summary statistics of seven seed traits**

|  | | Protein | Oil | Palmitic | Linoleic | | Oleic | Myristic | | Stearic |  | |
| --- | --- | --- | --- | --- | --- | --- | --- | --- | --- | --- | --- | --- |
| Minimum | | 33.02 | 24.4 | 17.58 | 27.81 | | 12.99 | 0.404 | | 1.77 |  | |
| Lower quartiles | | 38.03 | 30.49 | 20.67 | 56.24 | | 16.18 | 0.58 | | 2.3 |  | |
| Median | | 39.92 | 34.06 | 21.85 | 57.72 | | 17.34 | 0.6515 | | 2.44 |  | |
| Mean | | 40.53 | 33.55 | 21.64 | 57.57 | | 17.38 | 0.6537 | | 2.435 |  | |
| Upper quartiles | | 43.32 | 36.5 | 22.7 | 59.02 | | 18.55 | 0.73 | | 2.57 |  | |
| Maximum | | 48.35 | 41.52 | 25.56 | 63.84 | | 22.6 | 0.96 | | 3.14 |  | |
|  | | | | | | | | |  | | |  |
| **Accession ID** | **Accession name** | | | | | **Ecotype^a^** | | | **Species** | | | **Collection region** |
| RAD_25 | AnJiYan6Hao | | | | | CA | | | *G. hirsutum* | | | Former Soviet Union |
| RAD_167 | SuLian8911 | | | | | CA | | | *G. hirsutum* | | | Former Soviet Union |
| RAD_254 | r-3149 | | | | | OTH | | | *G. hirsutum* | | | Vietnam |
| RAD_255 | r4136 | | | | | OTH | | | *G. hirsutum* | | | Vietnam |
| RAD_249 | KuChe96518 | | | | | CA | | | *G. hirsutum* | | | Xinjiang, China |
| RAD_234 | Tai83158 | | | | | YR | | | *G. hirsutum* | | | Shanxi, China |
| RAD_231 | Ji91-22 | | | | | YR | | | *G. hirsutum* | | | Hebei, China |
| RAD_131 | Zhong961716 | | | | | YR | | | *G. hirsutum* | | | Henan, China |
| RAD_230 | SuLianMian116Xi | | | | | CA | | | *G. hirsutum* | | | Former Soviet Union |
| RAD_120 | JiA-7-8(33Xi) | | | | | YR | | | *G. hirsutum* | | | Hebei, China |
| RAD_27 | KuiTun80-2056W(XinLuZao-3Hao) | | | | | CA | | | *G. hirsutum* | | | Xinjiang, China |
| RAD_165 | MM-2 | | | | | US | | | *G. hirsutum* | | | United States |
| RAD_215 | ZhongMianSuo35 | | | | | YR | | | *G. hirsutum* | | | Henan, China |
| RAD_2 | BaZhou5628 | | | | | CA | | | *G. hirsutum* | | | Xinjiang, China |
| RAD_244 | Yun93Kang393 | | | | | YR | | | *G. hirsutum* | | | Shanxi, China |
| RAD_129 | Yun92Kang124 | | | | | YR | | | *G. hirsutum* | | | Shanxi, China |
| RAD_236 | JinKang157 | | | | | YR | | | *G. hirsutum* | | | Shanxi, China |
| RAD_239 | KuCheT94-4 | | | | | CA | | | *G. hirsutum* | | | Xinjiang, China |
| RAD_153 | ZiSeMeiMian | | | | | US | | | *G. hirsutum* | | | United States |
| RAD_326 | JiYuan12-13 | | | | | YR | | | *G. hirsutum* | | | Hebei, China |
| RAD_1 | 70-29-5 | | | | | YZR | | | *G. hirsutum* | | | Yunnan, China |
| RAD_290 | ZhongMianSuo16 | | | | | YR | | | *G. hirsutum* | | | Henan, China |
| RAD_229 | ZhongZi9103 | | | | | YR | | | *G. hirsutum* | | | Henan, China |
| RAD_241 | Liao96-23-30 | | | | | CA | | | *G. hirsutum* | | | Liaoning, China |
| RAD_128 | Shan960329-2Yuan3 | | | | | YR | | | *G. hirsutum* | | | Shaanxi, China |
| RAD_180 | PAR-51 | | | | | OTH | | | *G. hirsutum* | | | Pakistan |
| RAD_106 | CZA(70)33 | | | | | OTH | | | *G. hirsutum* | | | Austrilia |
| RAD_94 | ShanNong3Hao | | | | | YR | | | *G. hirsutum* | | | Shangdong, China |
| RAD_7 | ZhaoYang70Hao | | | | | CA | | | *G. hirsutum* | | | Liaoning, China |
| RAD_217 | SGKShiXuan321 | | | | | YR | | | *G. hirsutum* | | | Hebei, China |
| RAD_189 | RNX189 | | | | | YR | | | *G. hirsutum* | | | Henan, China |
| RAD_220 | BPA68 | | | | | OTH | | | *G. hirsutum* | | | Africa |
| RAD_130 | Zhong870203 | | | | | YR | | | *G. hirsutum* | | | Henan, China |
| RAD_190 | RNX190 | | | | | YR | | | *G. hirsutum* | | | Henan, China |
| RAD_270 | HanDan333 | | | | | YR | | | *G. hirsutum* | | | Hebei, China |
| RAD_70 | XinDaLing | | | | | YR | | | *G. hirsutum* | | | Henan, China |
| RAD_147 | ZhongYuanHAS-1 | | | | | YR | | | *G. hirsutum* | | | Henan, China |
| RAD_186 | L142-9 | | | | | OTH | | | *G. hirsutum* | | | Africa |
| RAD_85 | NaShangQuDaHua | | | | | YZR | | | *G. hirsutum* | | | Guizhou, China |
| RAD_169 | M-8124-1159 | | | | | US | | | *G. hirsutum* | | | United States |
| RAD_176 | ZhongMianSuo19Hao(Zhong7886) | | | | | YR | | | *G. hirsutum* | | | Henan, China |
| RAD_154 | LiaoMian-5Hao | | | | | CA | | | *G. hirsutum* | | | Liaoning, China |
| RAD_170 | AoSiv2 | | | | | OTH | | | *G. hirsutum* | | | Austrilia |
| RAD_32 | SuLian8908 | | | | | CA | | | *G. hirsutum* | | | Former Soviet Union |
| RAD_44 | ShanNong6Hao | | | | | YR | | | *G. hirsutum* | | | Shangdong, China |
| RAD_45 | ShiYuan638 | | | | | YR | | | *G. hirsutum* | | | Hebei, China |
| RAD_67 | DaLingMian69Hao | | | | | YZR | | | *G. hirsutum* | | | Hunan, China |
| RAD_73 | ZhongARR40681 | | | | | YR | | | *G. hirsutum* | | | Henan, China |
| RAD_82 | Lu21 | | | | | YR | | | *G. hirsutum* | | | Shangdong, China |
| RAD_83 | HuBeiSongZiDaLing | | | | | YZR | | | *G. hirsutum* | | | Hubei, China |
| RAD_86 | JiMian12Hao（HanDan177） | | | | | YR | | | *G. hirsutum* | | | Hebei, China |
| RAD_96 | XingTai79-11 | | | | | YR | | | *G. hirsutum* | | | Hebei, China |
| RAD_99 | YanCheng1115 | | | | | YZR | | | *G. hirsutum* | | | Jiangsu, China |
| RAD_102 | ZhengKang01-505 | | | | | YR | | | *G. hirsutum* | | | Henan, China |
| RAD_105 | ZhongArc-105 | | | | | YR | | | *G. hirsutum* | | | Henan, China |
| RAD_110 | N73DeltapineNGF | | | | | OTH | | | *G. hirsutum* | | | Austrilia |
| RAD_112 | Mei28114-313 | | | | | US | | | *G. hirsutum* | | | United States |
| RAD_116 | GK22 | | | | | YR | | | *G. hirsutum* | | | Hebei, China |
| RAD_117 | Han109 | | | | | YR | | | *G. hirsutum* | | | Hebei, China |
| RAD_143 | ZhongYuan911 | | | | | YR | | | *G. hirsutum* | | | Henan, China |
| RAD_181 | Sudan2 | | | | | OTH | | | *G. hirsutum* | | | Sudan |
| RAD_185 | ZhongArc-185 | | | | | YR | | | *G. hirsutum* | | | Henan, China |
| RAD_191 | Ao152 | | | | | OTH | | | *G. hirsutum* | | | Austrilia |
| RAD_202 | Yu17-202 | | | | | YR | | | *G. hirsutum* | | | Henan, China |
| RAD_207 | UA887(You) | | | | | US | | | *G. hirsutum* | | | United States |
| RAD_209 | DP33B | | | | | US | | | *G. hirsutum* | | | United States |
| RAD_213 | ST474 | | | | | US | | | *G. hirsutum* | | | United States |
| RAD_221 | Zhong9708-221 | | | | | YR | | | *G. hirsutum* | | | Henan, China |
| RAD_227 | HanDan568 | | | | | YR | | | *G. hirsutum* | | | Hebei, China |
| RAD_247 | HanDan109 | | | | | YR | | | *G. hirsutum* | | | Hebei, China |
| RAD_256 | KuKe310-5110 | | | | | OTH | | | *G. hirsutum* | | | Austrilia |
| RAD_260 | JiYuan55(91Han14) | | | | | YR | | | *G. hirsutum* | | | Hebei, China |
| RAD_293 | XinXiang89S-210 | | | | | YR | | | *G. hirsutum* | | | Henan, China |
| RAD_299 | ZhongMianSuo50 | | | | | YR | | | *G. hirsutum* | | | Henan, China |
| RAD_301 | Ji91-18 | | | | | YR | | | *G. hirsutum* | | | Hebei, China |
| RAD_51 | ZhongARR40682 | | | | | YR | | | *G. hirsutum* | | | Henan, China |
| RAD_104 | ZhongJi926 | | | | | YR | | | *G. hirsutum* | | | Henan, China |
| RAD_308 | ZhongArc-308 | | | | | YR | | | *G. hirsutum* | | | Henan, China |
| RAD_174 | Arcot-1 | | | | | US | | | *G. hirsutum* | | | United States |
| RAD_172 | ZhongZi04184 | | | | | YR | | | *G. hirsutum* | | | Henan, China |
| RAD_78 | RTN78 | | | | | YR | | | *G. hirsutum* | | | Henan, China |
| RAD_313 | ZhongAR-RNX313 | | | | | YR | | | *G. hirsutum* | | | Henan, China |
| RAD_52 | ZhongARNnXu | | | | | YR | | | *G. hirsutum* | | | Henan, China |
| RAD_277 | SuMian2Hao(XuZhou553) | | | | | YZR | | | *G. hirsutum* | | | Jiangsu, China |
| RAD_135 | ZhongZi640 | | | | | YR | | | *G. hirsutum* | | | Henan, China |
| RAD_242 | Yun93Kang354 | | | | | YR | | | *G. hirsutum* | | | Shanxi, China |
| RAD_281 | GaoYiMian | | | | | YZR | | | *G. hirsutum* | | | Jiangxi, China |
| RAD_195 | LuNong9648 | | | | | YR | | | *G. hirsutum* | | | Shangdong, China |
| RAD_311 | ZhongAR-RNX311 | | | | | YR | | | *G. hirsutum* | | | Henan, China |
| RAD_159 | GP137 | | | | | US | | | *G. hirsutum* | | | United States |
| RAD_222 | Zhong9708-222 | | | | | YR | | | *G. hirsutum* | | | Henan, China |
| RAD_79 | RTN79 | | | | | YR | | | *G. hirsutum* | | | Henan, China |
| RAD_48 | SuXu138(ZaoShu) | | | | | YZR | | | *G. hirsutum* | | | Jiangsu, China |
| RAD_13 | ShaMaoChangTao | | | | | YZR | | | *G. hirsutum* | | | Jiangsu, China |
| RAD_210 | GK20 | | | | | YR | | | *G. hirsutum* | | | Hebei, China |
| RAD_163 | Zhong89-1 | | | | | YR | | | *G. hirsutum* | | | Henan, China |
| RAD_164 | Miscot78-27 | | | | | US | | | *G. hirsutum* | | | United States |
| RAD_158 | Coker139 | | | | | US | | | *G. hirsutum* | | | United States |
| RAD_47 | SuXu137(ZaoShu) | | | | | YZR | | | *G. hirsutum* | | | Jiangsu, China |
| RAD_199 | SuTKH-1 | | | | | YZR | | | *G. hirsutum* | | | Jiangsu, China |
| RAD_141 | YongJi1Hao | | | | | YR | | | *G. hirsutum* | | | Shanxi, China |
| RAD_156 | Arcot436 | | | | | US | | | *G. hirsutum* | | | United States |
| RAD_300 | Acala SJ-5 | | | | | US | | | *G. hirsutum* | | | United States |
| RAD_142 | YongJi2Hao | | | | | YR | | | *G. hirsutum* | | | Shanxi, China |
| RAD_103 | Zhong2108 | | | | | YR | | | *G. hirsutum* | | | Henan, China |
| RAD_316 | ZhongAR681-316 | | | | | YR | | | *G. hirsutum* | | | Henan, China |
| RAD_149 | Zhong521 | | | | | YR | | | *G. hirsutum* | | | Henan, China |
| RAD_98 | XuZhouBanBanMian | | | | | YZR | | | *G. hirsutum* | | | Jiangsu, China |
| RAD_315 | ZhongArc-315 | | | | | YR | | | *G. hirsutum* | | | Henan, China |
| RAD_91 | Zhong5913-2 | | | | | YR | | | *G. hirsutum* | | | Henan, China |
| RAD_307 | Zhong507145 | | | | | YR | | | *G. hirsutum* | | | Henan, China |
| RAD_205 | Zhong4612YaH | | | | | YR | | | *G. hirsutum* | | | Henan, China |
| RAD_24 | Ji91-31 | | | | | YR | | | *G. hirsutum* | | | Hebei, China |
| RAD_111 | N74-250 | | | | | OTH | | | *G. hirsutum* | | | Austrilia |
| RAD_193 | AoL23/757 | | | | | OTH | | | *G. hirsutum* | | | Austrilia |
| RAD_89 | LuMian11Hao(3389) | | | | | YR | | | *G. hirsutum* | | | Shangdong, China |
| RAD_109 | Miscot7803-52 | | | | | US | | | *G. hirsutum* | | | United States |
| RAD_175 | ZhongMianSuo17Hao（Zhong117） | | | | | YR | | | *G. hirsutum* | | | Henan, China |
| RAD_257 | JiZi123（JiMian25） | | | | | YR | | | *G. hirsutum* | | | Hebei, China |
| RAD_30 | QingKang1Hao | | | | | YZR | | | *G. hirsutum* | | | Sichuan, China |
| RAD_81 | JiFeng197 | | | | | YR | | | *G. hirsutum* | | | Hebei, China |
| RAD_151 | YuMian2067 | | | | | YR | | | *G. hirsutum* | | | Henan, China |
| RAD_60 | NangFenDaTao | | | | | YR | | | *G. hirsutum* | | | Shanxi, China |
| RAD_107 | DES926 | | | | | US | | | *G. hirsutum* | | | United States |
| RAD_36 | YangFen31Hao | | | | | YR | | | *G. hirsutum* | | | Shangdong, China |
| RAD_203 | Zhong1276 | | | | | YR | | | *G. hirsutum* | | | Henan, China |
| RAD_237 | Ji85-3 | | | | | YR | | | *G. hirsutum* | | | Hebei, China |
| RAD_218 | SiMian2Hao | | | | | YZR | | | *G. hirsutum* | | | Jiangsu, China |
| RAD_56 | JiangSuDaTao | | | | | YZR | | | *G. hirsutum* | | | Jiangsu, China |
| RAD_287 | XinYan96-48 | | | | | YR | | | *G. hirsutum* | | | Henan, China |
| RAD_74 | ZhongARR40683 | | | | | YR | | | *G. hirsutum* | | | Henan, China |
| RAD_178 | HG-BR-8 | | | | | OTH | | | *G. hirsutum* | | | Unknown |
| RAD_62 | XiaoXianDaLing | | | | | YZR | | | *G. hirsutum* | | | Anhui, China |
| RAD_15 | Su7036YuanYuan | | | | | YZR | | | *G. hirsutum* | | | Jiangsu, China |
| RAD_224 | GP67 | | | | | US | | | *G. hirsutum* | | | United States |
| RAD_204 | Zhong31-204 | | | | | YR | | | *G. hirsutum* | | | Henan, China |
| RAD_17 | WanJiu828 | | | | | YZR | | | *G. hirsutum* | | | Anhui, China |
| RAD_166 | MeiD#1 | | | | | US | | | *G. hirsutum* | | | United States |
| RAD_322 | HongTao | | | | | YR | | | *G. hirsutum* | | | Henan, China |
| RAD_183 | ZhaDeMian | | | | | OTH | | | *G. hirsutum* | | | Chad |
| RAD_278 | HanDanChangRong | | | | | YR | | | *G. hirsutum* | | | Hebei, China |
| RAD_321 | Zhong2201 | | | | | YR | | | *G. hirsutum* | | | Henan, China |
| RAD_31 | Sha24-3 | | | | | CA | | | *G. hirsutum* | | | Xinjiang, China |
| RAD_38 | YaHuang9103 | | | | | YR | | | *G. hirsutum* | | | Henan, China |
| RAD_161 | GP83 | | | | | US | | | *G. hirsutum* | | | United States |
| RAD_55 | JiHan2HaoXuanXi | | | | | YR | | | *G. hirsutum* | | | Hebei, China |
| RAD_93 | XingTai6 | | | | | YR | | | *G. hirsutum* | | | Hebei, China |
| RAD_206 | ZhongR03 | | | | | YR | | | *G. hirsutum* | | | Henan, China |
| RAD_245 | DP410B | | | | | US | | | *G. hirsutum* | | | United States |
| RAD_282 | MSCO-12 | | | | | US | | | *G. hirsutum* | | | United States |
| RAD_187 | RNX187 | | | | | YR | | | *G. hirsutum* | | | Henan, China |
| RAD_69 | QiFengDaLing | | | | | YZR | | | *G. hirsutum* | | | Jiangsu, China |
| RAD_268 | Ji668 | | | | | YR | | | *G. hirsutum* | | | Hebei, China |
| RAD_49 | J02-247 | | | | | YZR | | | *G. hirsutum* | | | Jiangsu, China |
| RAD_285 | XuShiDaTao | | | | | YZR | | | *G. hirsutum* | | | Jiangsu, China |
| RAD_126 | Shan2800 | | | | | YR | | | *G. hirsutum* | | | Shaanxi, China |
| RAD_188 | RNX188 | | | | | YR | | | *G. hirsutum* | | | Henan, China |
| RAD_226 | Zhong932906 | | | | | YR | | | *G. hirsutum* | | | Henan, China |
| RAD_298 | ZhongMianSuo49 | | | | | YR | | | *G. hirsutum* | | | Henan, China |
| RAD_20 | E408 | | | | | YZR | | | *G. hirsutum* | | | Hubei, China |
| RAD_8 | Hu749513 | | | | | YZR | | | *G. hirsutum* | | | Jiangsu, China |
| RAD_323 | LangHuangF10(ZongXu) | | | | | YR | | | *G. hirsutum* | | | Henan, China |
| RAD_76 | ZhongArc-76 | | | | | YR | | | *G. hirsutum* | | | Henan, China |
| RAD_320 | Ari-320 | | | | | YR | | | *G. hirsutum* | | | Henan, China |
| RAD_28 | LiXianDaTao | | | | | YZR | | | *G. hirsutum* | | | Hunan, China |
| RAD_243 | GK99-1 | | | | | YR | | | *G. hirsutum* | | | Hebei, China |
| RAD_152 | 86-1（72-100） | | | | | YR | | | *G. hirsutum* | | | Hebei, China |
| RAD_150 | ZhongYuan9112 | | | | | YR | | | *G. hirsutum* | | | Henan, China |
| RAD_148 | ChangKangMian | | | | | YZR | | | *G. hirsutum* | | | Jiangsu, China |
| RAD_173 | Acala SJ-4 | | | | | US | | | *G. hirsutum* | | | United States |
| RAD_41 | JiMian11（CK） | | | | | YR | | | *G. hirsutum* | | | Hebei, China |
| RAD_61 | ZhongZi4480 | | | | | YR | | | *G. hirsutum* | | | Henan, China |
| RAD_261 | RTBaiXu | | | | | US | | | *G. hirsutum* | | | United States |
| RAD_122 | JCG94 | | | | | YZR | | | *G. hirsutum* | | | Jiangsu, China |
| RAD_118 | ZhongMianSuo32 | | | | | YR | | | *G. hirsutum* | | | Henan, China |
| RAD_317 | 99633 | | | | | YR | | | *G. hirsutum* | | | Henan, China |
| RAD_292 | ZhongMianSuo41(SGK9708) | | | | | YR | | | *G. hirsutum* | | | Henan, China |
| RAD_97 | XuZhou244 | | | | | YZR | | | *G. hirsutum* | | | Jiangsu, China |
| RAD_145 | ZhongYuan9115 | | | | | YR | | | *G. hirsutum* | | | Henan, China |
| RAD_146 | ZhongYuan9116 | | | | | YR | | | *G. hirsutum* | | | Henan, China |
| RAD_296 | LiaoMian19 | | | | | CA | | | *G. hirsutum* | | | Liaoning, China |
| RAD_211 | YuMian９Hao(YuZao1109) | | | | | YR | | | *G. hirsutum* | | | Henan, China |
| RAD_238 | Ji91-32 | | | | | YR | | | *G. hirsutum* | | | Hebei, China |
| RAD_284 | 353DaLingDi3Xi | | | | | YR | | | *G. hirsutum* | | | Hebei, China |
| RAD_179 | LAPAR45 | | | | | OTH | | | *G. hirsutum* | | | Brazil |
| RAD_58 | NeiHuangDaZi | | | | | YR | | | *G. hirsutum* | | | Henan, China |
| RAD_46 | SuLianMian21Xi(91-133) | | | | | CA | | | *G. hirsutum* | | | Former Soviet Union |
| RAD_113 | Qik | | | | | US | | | *G. hirsutum* | | | United States |
| RAD_312 | ZhongG5 | | | | | YR | | | *G. hirsutum* | | | Henan, China |
| RAD_194 | JiA-1-7（33Xi） | | | | | YR | | | *G. hirsutum* | | | Hebei, China |
| RAD_100 | Yun3060 | | | | | YR | | | *G. hirsutum* | | | Shanxi, China |
| RAD_14 | SiChang2Xi | | | | | YZR | | | *G. hirsutum* | | | Jiangsu, China |
| RAD_123 | Han8944 | | | | | YR | | | *G. hirsutum* | | | Hebei, China |
| RAD_133 | ZhongZhiBD27 | | | | | YR | | | *G. hirsutum* | | | Hebei, China |
| RAD_84 | GP70 | | | | | US | | | *G. hirsutum* | | | United States |
| RAD_275 | QinYuan4Hao(QinYuan91406) | | | | | YR | | | *G. hirsutum* | | | Shaanxi, China |
| RAD_228 | ZhongZi9102 | | | | | YR | | | *G. hirsutum* | | | Henan, China |
| RAD_10 | Jin444 | | | | | CA | | | *G. hirsutum* | | | Liaoning, China |
| RAD_264 | Lu458 | | | | | YR | | | *G. hirsutum* | | | Shangdong, China |
| RAD_248 | Liao823-834-23 | | | | | CA | | | *G. hirsutum* | | | Liaoning, China |
| RAD_9 | HuaiMian4Hao | | | | | YZR | | | *G. hirsutum* | | | Anhui, China |
| RAD_57 | KangSanXingDaTao | | | | | YZR | | | *G. hirsutum* | | | Sichuan, China |
| RAD_75 | ZhongR773-75 | | | | | YR | | | *G. hirsutum* | | | Henan, China |
| RAD_66 | DaLingMian | | | | | YR | | | *G. hirsutum* | | | Liaoning, China |
| RAD_274 | DaZeMian | | | | | YR | | | *G. hirsutum* | | | Shangdong, China |
| RAD_92 | Zhong85271 | | | | | YR | | | *G. hirsutum* | | | Henan, China |
| RAD_286 | ChengDingDaLing | | | | | YR | | | *G. hirsutum* | | | Hebei, China |
| RAD_119 | ZhongAR40772 | | | | | YR | | | *G. hirsutum* | | | Henan, China |
| RAD_65 | DaLingFuZiMian | | | | | YZR | | | *G. hirsutum* | | | Jiangsu, China |
| RAD_34 | Tu83-161 | | | | | CA | | | *G. hirsutum* | | | Xinjiang, China |
| RAD_115 | MeiG-84 | | | | | US | | | *G. hirsutum* | | | United States |
| RAD_219 | SuLianMian91Xi | | | | | CA | | | *G. hirsutum* | | | Former Soviet Union |
| RAD_269 | XuZhou261 | | | | | YZR | | | *G. hirsutum* | | | Jiangsu, China |
| RAD_198 | SuQ1 | | | | | YZR | | | *G. hirsutum* | | | Jiangsu, China |
| RAD_4 | Bao6722 | | | | | YR | | | *G. hirsutum* | | | Hebei, China |
| RAD_306 | Zhong2220 | | | | | YR | | | *G. hirsutum* | | | Henan, China |
| RAD_212 | ZhongMianSuo27 | | | | | YR | | | *G. hirsutum* | | | Henan, China |
| RAD_192 | AoC | | | | | OTH | | | *G. hirsutum* | | | Austrilia |
| RAD_329 | ZhongR773-329 | | | | | YR | | | *G. hirsutum* | | | Henan, China |
| RAD_121 | JCG59 | | | | | YZR | | | *G. hirsutum* | | | Jiangsu, China |
| RAD_132 | ZhongZhiBD13 | | | | | YR | | | *G. hirsutum* | | | Hebei, China |
| RAD_325 | ZhongZi10Hao | | | | | YR | | | *G. hirsutum* | | | Henan, China |
| RAD_177 | Su08B2-177 | | | | | YR | | | *G. hirsutum* | | | Henan, China |
| RAD_134 | ZhongZhiBD89 | | | | | YR | | | *G. hirsutum* | | | Hebei, China |
| RAD_6 | ChangRong67-12 | | | | | YZR | | | *G. hirsutum* | | | China |
| RAD_253 | DP2156 | | | | | US | | | *G. hirsutum* | | | United States |
| RAD_29 | MianYang73-39 | | | | | YZR | | | *G. hirsutum* | | | Sichuan, China |
| RAD_294 | M11 | | | | | OTH | | | *G. hirsutum* | | | Pakistan |
| RAD_108 | GP93 | | | | | US | | | *G. hirsutum* | | | United States |
| RAD_318 | SuYuan04-162 | | | | | YZR | | | *G. hirsutum* | | | Jiangsu, China |
| RAD_101 | Yun92A-260 | | | | | YR | | | *G. hirsutum* | | | Shanxi, China |
| RAD_314 | ZhongR773-314 | | | | | YR | | | *G. hirsutum* | | | Henan, China |
| RAD_95 | Si168 | | | | | YZR | | | *G. hirsutum* | | | Jiangsu, China |
| RAD_262 | MSCO-11 | | | | | US | | | *G. hirsutum* | | | United States |
| RAD_114 | MeiG-82 | | | | | US | | | *G. hirsutum* | | | United States |
| RAD_155 | Arcot402bne | | | | | US | | | *G. hirsutum* | | | United States |
| RAD_291 | LineF | | | | | US | | | *G. hirsutum* | | | United States |
| RAD_125 | Shan2747 | | | | | YR | | | *G. hirsutum* | | | Shaanxi, China |
| RAD_259 | ZhongYuanHST1 | | | | | YR | | | *G. hirsutum* | | | Henan, China |
| RAD_37 | ZaoShuChangRong7 | | | | | US | | | *G. hirsutum* | | | United States |
| RAD_137 | Liao4853 | | | | | CA | | | *G. hirsutum* | | | Liaoning, China |
| RAD_233 | KuChe93551 | | | | | CA | | | *G. hirsutum* | | | Xinjiang, China |
| RAD_252 | Ji91-33 | | | | | YR | | | *G. hirsutum* | | | Hebei, China |
| RAD_279 | JiHaiLu6(91Han6) | | | | | YR | | | *G. hirsutum* | | | Hebei, China |
| RAD_136 | Liao4835 | | | | | CA | | | *G. hirsutum* | | | Liaoning, China |
| RAD_309 | ZhongR773-309 | | | | | YR | | | *G. hirsutum* | | | Henan, China |
| RAD_77 | ZhongAR683-77 | | | | | YR | | | *G. hirsutum* | | | Henan, China |
| RAD_327 | Ari-327 | | | | | YR | | | *G. hirsutum* | | | Henan, China |
| RAD_72 | ZhongＲ773-72 | | | | | YR | | | *G. hirsutum* | | | Henan, China |
| RAD_251 | Zhong07 | | | | | YR | | | *G. hirsutum* | | | Henan, China |
| RAD_225 | ZhaDe3Hao | | | | | OTH | | | *G. hirsutum* | | | Chad |
| RAD_138 | LiaoMian16 | | | | | CA | | | *G. hirsutum* | | | Liaoning, China |
| RAD_139 | LiaoMian17 | | | | | CA | | | *G. hirsutum* | | | Liaoning, China |
| RAD_310 | ZhongR773-310 | | | | | YR | | | *G. hirsutum* | | | Henan, China |
| RAD_54 | S-050031 | | | | | CA | | | *G. hirsutum* | | | Xinjiang, China |
| RAD_124 | KangHuangWei164 | | | | | YR | | | *G. hirsutum* | | | Henan, China |
| RAD_22 | Han8959 | | | | | YR | | | *G. hirsutum* | | | Hebei, China |
| RAD_171 | Mei8123 | | | | | US | | | *G. hirsutum* | | | United States |
| RAD_182 | FeiZhouE40 | | | | | OTH | | | *G. hirsutum* | | | Africa |
| RAD_280 | PD6186 | | | | | US | | | *G. hirsutum* | | | United States |
| RAD_68 | MeiFuDaLing | | | | | YR | | | *G. hirsutum* | | | Henan, China |
| RAD_140 | LiaoMian18 | | | | | CA | | | *G. hirsutum* | | | Liaoning, China |
| RAD_250 | Bu3363 | | | | | US | | | *G. hirsutum* | | | United States |
| RAD_235 | Ji91-12 | | | | | YR | | | *G. hirsutum* | | | Hebei, China |
| RAD_168 | AC239 | | | | | US | | | *G. hirsutum* | | | United States |
| RAD_63 | Acala(DaLing)B | | | | | US | | | *G. hirsutum* | | | United States |
| RAD_208 | AiZiMian927 | | | | | US | | | *G. hirsutum* | | | United States |
| RAD_127 | Shan3184 | | | | | YR | | | *G. hirsutum* | | | Shaanxi, China |
| RAD_271 | QinLi514 | | | | | YR | | | *G. hirsutum* | | | Shaanxi, China |
| RAD_328 | BaZhou7416 | | | | | CA | | | *G. hirsutum* | | | Xinjiang, China |
| RAD_18 | Liao61107 | | | | | CA | | | *G. hirsutum* | | | Liaoning, China |
| RAD_297 | AcalaSJ-1-9 | | | | | US | | | *G. hirsutum* | | | United States |
| RAD_324 | SuMian9108 | | | | | YZR | | | *G. hirsutum* | | | Anhui, China |
| RAD_90 | Yu284 | | | | | YR | | | *G. hirsutum* | | | Henan, China |
| RAD_184 | Acala SJ-1 | | | | | US | | | *G. hirsutum* | | | United States |
| RAD_59 | NongJiu | | | | | US | | | *G. hirsutum* | | | United States |
| RAD_21 | Han8901 | | | | | YR | | | *G. hirsutum* | | | Hebei, China |
| RAD_263 | upland | | | | | US | | | *G. hirsutum* | | | United States |
| RAD_289 | ZhongZi9196（JiGuang） | | | | | YR | | | *G. hirsutum* | | | Henan, China |
| RAD_265 | YuMian19 | | | | | YR | | | *G. hirsutum* | | | Henan, China |
| RAD_160 | GP138 | | | | | US | | | *G. hirsutum* | | | United States |
| RAD_267 | EKangMian9Hao | | | | | YZR | | | *G. hirsutum* | | | Hubei, China |
| RAD_266 | WangJiangChangRongMian | | | | | YZR | | | *G. hirsutum* | | | Anhui, China |
| RAD_196 | MeiF-18 | | | | | US | | | *G. hirsutum* | | | United States |
| RAD_144 | ZhongYuan9114 | | | | | YR | | | *G. hirsutum* | | | Henan, China |
| RAD_319 | SuYuan04-129 | | | | | YZR | | | *G. hirsutum* | | | Jiangsu, China |
| RAD_288 | YuMian2Hao(YuWu302) | | | | | YR | | | *G. hirsutum* | | | Henan, China |
| RAD_272 | 601ChangRongMian | | | | | YZR | | | *G. hirsutum* | | | Jiangsu, China |
| RAD_3 | Bao6716 | | | | | YR | | | *G. hirsutum* | | | Hebei, China |
| RAD_200 | SuYuan7235 | | | | | YZR | | | *G. hirsutum* | | | Jiangsu, China |
| RAD_16 | SuNong6Hao | | | | | YZR | | | *G. hirsutum* | | | Jiangsu, China |
| RAD_42 | Jing55168 | | | | | YZR | | | *G. hirsutum* | | | Hubei, China |
| RAD_330 | Acala1517-2 | | | | | US | | | *G. hirsutum* | | | United States |
| RAD_43 | KuiTunXi96-167 | | | | | CA | | | *G. hirsutum* | | | Xinjiang, China |
| RAD_35 | XiangMian2Hao | | | | | YZR | | | *G. hirsutum* | | | Hunan, China |
| RAD_19 | ChangRongMuZiMian | | | | | YR | | | *G. hirsutum* | | | Henan, China |
| RAD_33 | Tu188 | | | | | CA | | | *G. hirsutum* | | | Xinjiang, China |
| RAD_80 | DaTaoMian(Yu) | | | | | YR | | | *G. hirsutum* | | | Henan, China |
| RAD_283 | 0102X-10-1 | | | | | CA | | | *G. hirsutum* | | | Xinjiang, China |
| RAD_197 | MeiF-19 | | | | | US | | | *G. hirsutum* | | | United States |
| RAD_26 | Jing55263 | | | | | YZR | | | *G. hirsutum* | | | Hubei, China |
| RAD_50 | J02-508 | | | | | YZR | | | *G. hirsutum* | | | Jiangsu, China |
| RAD_273 | LuMianYan21(Lu1138) | | | | | YR | | | *G. hirsutum* | | | Shangdong, China |
| RAD_71 | XinZhouDaLing | | | | | YZR | | | *G. hirsutum* | | | Hubei, China |
| RAD_53 | S-050019 | | | | | CA | | | *G. hirsutum* | | | Xinjiang, China |
| RAD_201 | ShenMian5Hao | | | | | YR | | | *G. hirsutum* | | | Shaanxi, China |
| RAD_12 | KuCheT94-6 | | | | | CA | | | *G. hirsutum* | | | Xinjiang, China |
| RAD_88 | LinQing201 | | | | | YR | | | *G. hirsutum* | | | Shangdong, China |
| RAD_23 | HuaZhong91-0102 | | | | | YZR | | | *G. hirsutum* | | | Hubei, China |
| RAD_276 | ZhengZhouZhangRongMian | | | | | YR | | | *G. hirsutum* | | | Henan, China |
| RAD_11 | KuCheT94-1 | | | | | CA | | | *G. hirsutum* | | | Xinjiang, China |
| RAD_5 | ZhangDe184 | | | | | YR | | | *G. hirsutum* | | | Henan, China |
| RAD_87 | KuChe96486 | | | | | CA | | | *G. hirsutum* | | | Xinjiang, China |
| RAD_39 | ChuanGaoYiFen58 | | | | | YZR | | | *G. hirsutum* | | | Sichuan, China |
| RAD_223 | TM1-IPR | | | | | YR | | | *G. hirsutum* | | | Henan, China |
| RAD_162 | GP95 | | | | | US | | | *G. hirsutum* | | | United States |
